# Large-scale functional neural network correlates of response inhibition: an fMRI meta-analysis

**DOI:** 10.1007/s00429-017-1443-x

**Published:** 2017-05-27

**Authors:** Ruibin Zhang, Xiujuan Geng, Tatia M. C. Lee

**Affiliations:** 1 Laboratory of Neuropsychology, The University of Hong Kong, Rm 656, Jockey Club Tower, Pokfulam Road, Hong Kong, Hong Kong; 2 Laboratory of Cognitive Affective Neuroscience, The University of Hong Kong, Hong Kong, Hong Kong; 3 The State Key Laboratory of Brain and Cognitive Sciences, The University of Hong Kong, Hong Kong, Hong Kong; 4 Institute of Clinical Neuropsychology, The University of Hong Kong, Hong Kong, Hong Kong

**Keywords:** Multilevel kernel density analysis, Meta-analysis, fMRI, Interference resolution, Action restrain

## Abstract

An influential hypothesis from the last decade proposed that regions within the right inferior frontal cortex of the human brain were dedicated to supporting response inhibition. There is growing evidence, however, to support an alternative model, which proposes that neural areas associated with specific inhibitory control tasks co-exist as common network mechanisms, supporting diverse cognitive processes. This meta-analysis of 225 studies comprising 323 experiments examined the common and distinct neural correlates of cognitive processes for response inhibition, namely interference resolution, action withholding, and action cancellation. Activation coordinates for each subcategory were extracted using multilevel kernel density analysis (MKDA). The extracted activity patterns were then mapped onto the brain functional network atlas to derive the common (i.e., process-general) and distinct (i.e., domain-oriented) neural network correlates of these processes. Independent of the task types, activation of the right hemispheric regions (inferior frontal gyrus, insula, median cingulate, and paracingulate gyri) and superior parietal gyrus was common across the cognitive processes studied. Mapping the activation patterns to a brain functional network atlas revealed that the fronto-parietal and ventral attention networks were the core neural systems that were commonly engaged in different processes of response inhibition. Subtraction analyses elucidated the distinct neural substrates of interference resolution, action withholding, and action cancellation, revealing stronger activation in the ventral attention network for interference resolution than action inhibition. On the other hand, action withholding/cancellation primarily engaged the fronto-striatal circuit. Overall, our results suggest that response inhibition is a multidimensional cognitive process involving multiple neural regions and networks for coordinating optimal performance. This finding has significant implications for the understanding and assessment of response inhibition.

**Electronic supplementary material:**

The online version of this article (doi:10.1007/s00429-017-1443-x) contains supplementary material, which is available to authorized users.

## Introduction

Response inhibition refers to the ability to suppress automatic actions or behaviors that are not appropriate or no longer adaptive to the situation (Aron 2007; Goldman-Rakic et al. 1996). Dysfunctional response inhibition is a core symptom observed in various mental disorders, including attention deficit hyperactivity disorder (Schachar et al. 2007), schizophrenia (Vercammen et al. 2012), and depression (Palazidou 2012), as well as learning difficulties (Eickhoff et al. 2009) and behavioral problems (Liu et al. 2012). Thus, understanding of response inhibition and its neural correlates in the healthy population could not only enhance our knowledge of cognitive neural networks but also offer a unique platform for identifying and clinically addressing neural markers of dysfunctional response inhibition.

### Measurement of response inhibition

Paradigms such as the stroop, Flanker, Simon, stimulus response compatibility (SRC), antisaccade tasks, stop signal, and Go/NoGo tasks, which require a response to a target stimulus among irrelevant/distracting stimuli, are commonly regarded as paradigms that involve inhibitory action control (Nigg 2000) and are commonly used for the investigation of response inhibition mechanisms (Nee et al. 2007; van Velzen et al. 2014; Swick et al. 2011; Stahl et al. 2014; Nigg 2000). In stop signal and Go/NoGo tasks, increased automatic tendency to initiate a particular motor response is induced through a higher frequency of go trials than inhibition trials (i.e., NoGo or stop). The resultant action bias has to be suppressed when the inhibition signal is presented during NoGo or stop trials. In the Go/NoGo task, participants have to withhold a prepotent but not yet initiated motor response, whereas the stop signal task cancels an already initiated motor response. In the other tasks, which can be organized under the term “incongruency tasks” (Cieslik et al. 2015), a given stimulus dimension interferes with relevant stimuli and/or response information, thereby affecting responses to the relevant information.

These tasks all require cognitive control over a predominant response tendency and the context-dependent initiation of an appropriate behavioral alternative—that is, either to initiate an alternative, nondominant response, or to not respond at all. Given the similarities and differences across these tasks, it is important to explore whether their neural correlates are shared, distinct, or both. To achieve this goal, we categorized these paradigms into three subcategories: inference resolution, action withholding, and action cancellation (Sebastian et al. 2013b; Stahl et al. 2014; Friedman and Miyake 2004; Hasher et al. 2007). Interference resolution is the process of selecting information with regard to its relevance to an ongoing task and suppressing the processing of irrelevant information (Yarkoni et al. 2010). This process is usually tested by the Stroop, Simon, Flanker, SRC, and antisaccade tasks (Stahl et al. 2014; van Velzen et al. 2014). Comparatively, action withholding is commonly assessed using the Go/NoGo task. The stop signal task is conceptualized as an action cancellation task.

### Neural correlates of response inhibition

**Fig. 1:**
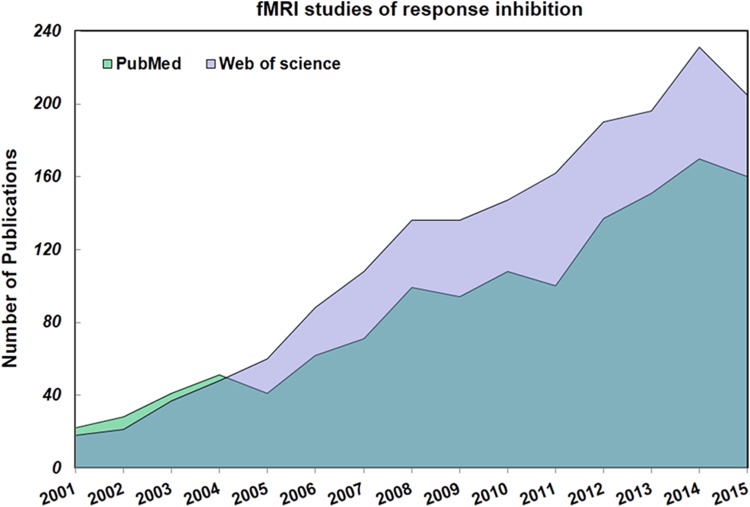
Number of studies investigating the neural correlates of response inhibition using fMRI over the last 15 years (1 Jan. 2001–31 Dec. 2015). Two databases—PubMed and Web of Science—were searched using the keywords “response inhibition” and “fMRI”

Over the past 15 years, there has been a dramatic increase in the number of studies examining neural correlates of response inhibition using functional magnetic resonance imaging (fMRI) (refer to Fig. 1). Based on the results of these studies, several different models of inhibitory control have been put forward (e.g., Aron et al. 2004, 2014; Swick and Chatham 2014; Van Belle et al. 2014; Zandbelt et al. 2013; Hampshire et al. 2010, 2015). Among these models, those proposed by Aron and Hampshire are arguably the most prominent and influential. Aron and colleagues proposed that response inhibition activated a module mainly in the right lateralized frontal brain areas, in which the inferior frontal gyrus (IFG) was the key component. During intact response inhibition, inhibitory neural stop signals may then be sent from these frontal regions to the motor cortices through cortico-striatal–thalamic–cortical projections (Aron 2011; Chambers et al. 2009). Hampshire’s group, on the other hand, suggested that response inhibition was one example of a broader class of control processes. These control processes are supported by the same set of fronto-parietal networks that exert control by modulating local lateral inhibition processes, which occur ubiquitously throughout the cortex (Hampshire et al. 2010). Thus, instead of focusing on how a specific brain region and its connection pathways may support response inhibition, understanding the neural basis of behavioral control may require a more holistic approach that considers how common network mechanisms support diverse cognitive processes (Hampshire and Sharp 2015).

The previous studies have indicated common regions activated by response inhibition tasks, including those correlates comprising the fronto-parietal network, specifically the IFG, pre-SMA, and parietal regions. For example, Sebastian et al. (2013b), using hybrid tasks combining Simon, Go/NoGo, and stop signals, demonstrated that areas such as the IFG and the bilateral parietal regions showed activation across all tasks. Nonetheless, unique neural networks for the three subcategories of processes underpinning response inhibition have also been suggested (Rubia et al. 2001; Sebastian et al. 2013b; Chevrier et al. 2007; van Velzen et al. 2014; Sebastian et al. 2013a). For example, the Simon task (interference resolution) was found to stimulate the pre-motor and parietal regions to a greater extent than action cancellation (stop signals), whereas action cancellation was found to elicit stronger activation in the bilateral posterior inferior frontal gyrus/insula and the right striatum than action withholding (Go/NoGo) (Sebastian et al. 2013b). Although the above findings provide important preliminary evidence of the potential common and distinct neural underpinnings of different response inhibition tasks, the majority of studies have either explored only a single response inhibition task in small samples with limited statistical power or were challenging to compare, because they employed various different stimuli (e.g., visual, auditory). We are, therefore, hindered from drawing complete conclusions on the common and distinct neural correlates of the subcategory of processes for response inhibition (Swick et al. 2011; Logan et al. 2015). An understanding of the common and unique neural correlates of the subcategories of cognitive processes associated with response inhibition provides critical insight into early detection, diagnostic accuracy, and treatment targets of clinical disorders afflicted by dysfunctional response inhibition (Aron 2011; Chambers et al. 2009). Meta-analytic pooling of all related studies is a powerful statistical tool that combines data sets from a collection of similar studies to obtain a more accurate and robust estimate of the effect size of a given phenomenon (Fox et al. 2014), and thus may serve as a promising approach to studying the common and distinct neural correlates of the three subcategories of processes associated with response inhibition.

Furthermore, investigation of network correlated activations may provide system-level interpretations as to what neural mechanisms underlie response inhibition (Damoiseaux et al. 2006; Smith et al. 2009; Erika-Florence et al. 2014). Indeed, studies have suggested that response inhibition is correlated with the connections between the cingulo-opercular network (salience network) and fronto-parietal network, and that default mode network activity might be actively downregulated by an inhibitory module within the fronto-parietal network (Leech et al. 2011). Aberrant network interactions are proposed to lead to deficits in response inhibition (Hampshire and Sharp 2015; Jilka et al. 2014). A large-scale network approach will help to clarify these speculations and provide system-level understanding about the working principle of response inhibition (Leech and Sharp 2014). Given that response inhibition is subserved by a large-scale distributed system that includes the bilateral cortical and subcortical regions (Swick et al. 2011), conceiving of a large-scale network rather than the classical region-based functional-anatomy analysis will be more suitable for this purpose (Woo et al. 2014; Hampshire and Sharp 2015).

In summary, the overall objective of this study is to provide a quantitative summary of the major findings across studies on response inhibition, on the basis of previously reported neural network parcellations (Yeo et al. 2011; Choi et al. 2012). Specifically, we aimed to (1) examine activation patterns across all studies using multilevel kernel density analysis (MKDA); (2) analyze the brain functional network correlates responsible for interference resolution, action withholding, and action cancellation; and (3) identify the common and primary neural network correlates for each subcategory through conjunction and subtraction analyses, respectively.

## Methods and materials

### Literature search and selection criteria

**Fig. 2:**
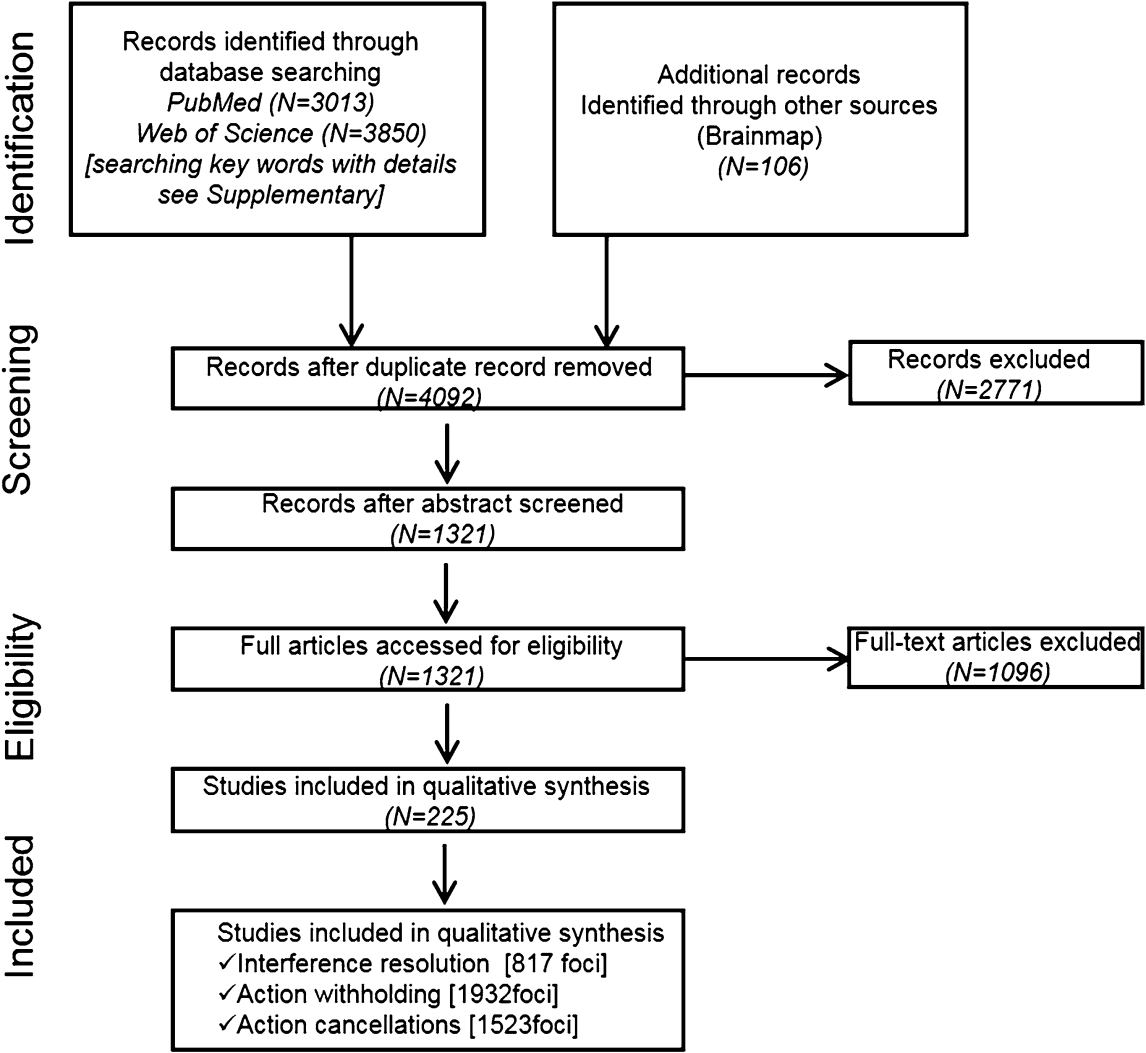
Flowchart of searching and selection of literature in response inhibition

Two online citation indexing services—PubMed and Web of Science—were searched. Keywords including “fMRI” with “response inhibition”, “inhibitory control”, “interference resolution”, “action withholding”, “action cancellation”, “stop signal”, “stopping”, “go nogo”, “action restraint”, or “countermanding” were used to retrieve relevant literature published prior to Dec. 31, 2015 (for detailed search results, please see Table S1 of the Supplementary Materials). We also searched the BrainMap database using Sleuth (http://brainmap.org/) within the imaging modality of “fMRI” and the behavioral domain of “action inhibition” and obtained 106 papers. All articles were pooled into a database, and redundant entries were eliminated, yielding 4092 reports. We applied the following exclusion criteria to eliminate articles that were not directly relevant to this study: (1) nonoriginal studies (e.g., review articles), (2) studies that did not report results in standard stereotactic coordinate space (either Talairach or the Montreal Neurological Institute, MNI), (3) studies that were purely based on region of interest (ROI) analysis (e.g., using anatomical masks or coordinates from other studies), (4) analyses applying methods other than nonlinear modeling, e.g., multi-variate pattern analysis (MVPA), (5) studies with sample size below five and/or age range of participants outside 18–65 years, (6) studies on atypical populations whose brain functions may have deviated from those of healthy adults and those in which results for healthy controls were not reported separately, (7) pharmacological or training-related studies, and (8) single-sex studies. A total of 225 full articles were included in the current meta-analysis (Demographic data see Table S2 of Supplementary Materials). The detailed searching and selection procedures are shown in Fig. 2.

### Experiment categorization

#### Interference resolution

Interference resolution is the process of selecting information with regard to its relevance to an ongoing task and suppressing the processing of irrelevant information (Yarkoni et al. 2010). This process can be captured by stimulus response incompatibility tasks, such as the Stroop, Simon, Flanker, and Wisconsin Card Sorting Test paradigms (Stahl et al. 2014; van Velzen et al. 2014). We examined the brain activation profiles of participants who performed these tasks to understand the neural activity underpinning interference resolution. On the behavioral level, for example, Stroop and Flanker are similar in that both require the ability to control stimulus-related interference as well as the ability to control response-related interference (Stahl et al. 2014). On the neural level, for example, Liu et al. (2004) found that both tasks activated brain regions that serve as a source of attentional control, such as the dorsolateral prefrontal cortex, and posterior regions that are sites of attentional control, such as the visual processing stream (the middle occipital and inferior temporal cortices). Our analyses suggested that no specific paradigms disproportionately altered the results (Table 5 and Fig. S1 in Supplementary Materials). Thus, 50 articles consisting of 68 experiments with 817 foci were included to explore the neural correlates of interference resolution. The characteristics of each study are listed in Table S2 of the Supplementary Materials.

#### Action withholding

Go/NoGo paradigms require individuals to rapidly respond to pre-defined “Go” stimuli while withholding responses to pre-defined “NoGo” stimuli presented in random sequence, and thus, they are classified as eliciting action withholding. A measure of action withholding is the proportion of inhibited stimuli (“NoGo” trials) relative to noninhibited stimuli (Go trials) (van Velzen et al. 2014). The effects of such “NoGo” frequency should be considered as low frequency. “NoGo” signals have been found to engage significant attention resources (Criaud and Boulinguez 2013). Different contrast conditions for Go/NoGo tasks exist, e.g., “NoGo” trials vs. “Go” trials and “NoGo” vs. fixation cross as baseline (e.g., van Rooij et al. 2015) or a low-level baseline (e.g., Claus and Hendershot 2015). We compared experiments with high frequency of “NoGo” stimuli to those with low frequency (i.e., equal to 50% and less than 50%, respectively). We found that the network correlate distributions of activated areas were not significantly different (Table S6 and S7 in Supplementary Materials). Thus, 117 articles using the Go/NoGo paradigm and comprising 147 contrasts were employed to identify the action withholding-related activation patterns.

#### Action cancellation

We classified stop signal tasks as those that trigger action cancellation (Aron et al. 2004; Schachar et al. 2007). Stop signal paradigms consist of two concurrent tasks, i.e., a go task and a stop task. In a stop signal task, individuals are instructed to make rapid choices about target stimuli. In some trials, a second stimulus (e.g., auditory) is presented shortly after the target, and individuals need to cancel their response, which has often been initiated already. Thus, the demand for response inhibition is high, because the stop signals are randomly presented in an array of go trials. Participants are instructed to cancel their initiated actions when the stop signals are presented. The design of the task is to ensure inhibition of approximately 50% of the go responses following a stop signal. Since the most appropriate comparison conditions for stop signal tasks have been debated in the literature (Boehler et al. 2010; Swick et al. 2011), we did a Chi-square test and found that the network correlates distribution between all contrasts, and contrasts that only included the stop–go paradigm did not differ at a significant level (detailed condition comparisons of each stop signal task and the results are presented in Table S8 in the Supplementary Materials). Seventy-three related articles with 108 stop signal experiments were included in this meta-analysis to summarize the activation patterns of action cancellation.

### Data extraction

We extracted the following information from each study: authors, year of publication, sample size, experimental design, paradigms and task contrasts (including the frequency of the presented inhibitory stimuli), field strength of the MRI scanner, and cluster coordinates in the MNI or Talairach space (Table S2 in Supplementary Materials). As the MNI/Talairach coordinate bias associated with reference frame (position and orientation) and scale (brain size) can be substantially reduced using the best-fit tal2icbm transform (Lancaster et al. 2007), coordinates that were not reported in the MNI space were transformed using the Lancaster transformation.

### Multilevel kernel density analysis (MKDA)

We conducted meta-analyses using the MKDA (Wager et al. 2007) toolbox (http://wagerlab.colorado.edu) to identify brain regions activated during response inhibition. Peak effect coordinates from each study were convolved with a spherical kernel (*r* = 5 mm) and threshold to obtain an indicator map, with a value of one indicating a significant effect in the neighborhood and a value of zero indicating no significant effect. The density of the effect was computed by averaging the indicator maps weighted by the study sample size, and the resulting density maps showed the proportion of studies in which activation was observed within 5 mm of each voxel. The family wise error (FWE) rate was estimated with a Monte Carlo simulation to correct for multiple comparisons, and a natural null hypothesis was that the “activated” regions are randomly distributed throughout the brain. Thus, the reported meta-analytic results in this study represent consistently activated regions across studies: regions in which significant activations were observed in the local neighborhood by more studies than would be expected by chance (*p* < 0.05, FWE corrected across the entire brain). The MKDA was performed for characterizing brain activation patterns. First, we identified those brain regions that showed significant convergence across 225 studies comprising 3453 foci from 323 contrasts. Then, three additional MKDA analyses were conducted for the specific activations produced by the three subcategories: interference resolution, action withholding, and action cancellation. For interference resolution, 817 foci from 68 contrasts were included. The analyses for action withholding consisted of 1932 foci from 147 contrasts, and the analyses for action cancellation included 1523 foci from 108 contrasts. The same statistical analyses and thresholding approaches were applied for all meta-analyses in this study. Three subtraction analyses were conducted to capture the selectively or preferentially activated brain regions for the different classifications of responses: interference resolution vs. action withholding, interference resolution vs. action cancellation, and action withholding vs. action cancellation.

### Activation patterns from the functional network correlate perspective

To examine the related neural network correlates, voxels significantly activated by response inhibition and its sub-processes were overlaid onto seven commonly referenced brain functional network correlates covering the cerebral cortex and striatum (Yeo et al. 2011; Choi et al. 2012): the fronto-parietal network (FPN), dorsal attention network (DAN), ventral attention network (VAN), somatomotor network (SMN), visual network (VN), affective network (AFN), and default mode network (DMN). Chi-square tests were performed to contrast the proportions of the activated voxels in the seven network correlates distributed among interference resolution, action withholding, and action cancellation.

### Effect of contrast numbers

Action withholding (147 experiments of Go/NoGo tasks) and action cancellation (108 experiments of stop signal tasks) included approximately two times the number of experiments as interference resolution (68 experiments). Random selections of 68 contrasts from action withholding and action cancellation were performed to test the effect of the experiment numbers. We repeated the MKDA analysis using the same settings, and Chi-square analyses were performed to compare the activated voxels in the seven network correlates to those of our main analyses and the random selected studies.

## Results

### Meta-analysis of all included response inhibition experiments

**Fig. 3:**
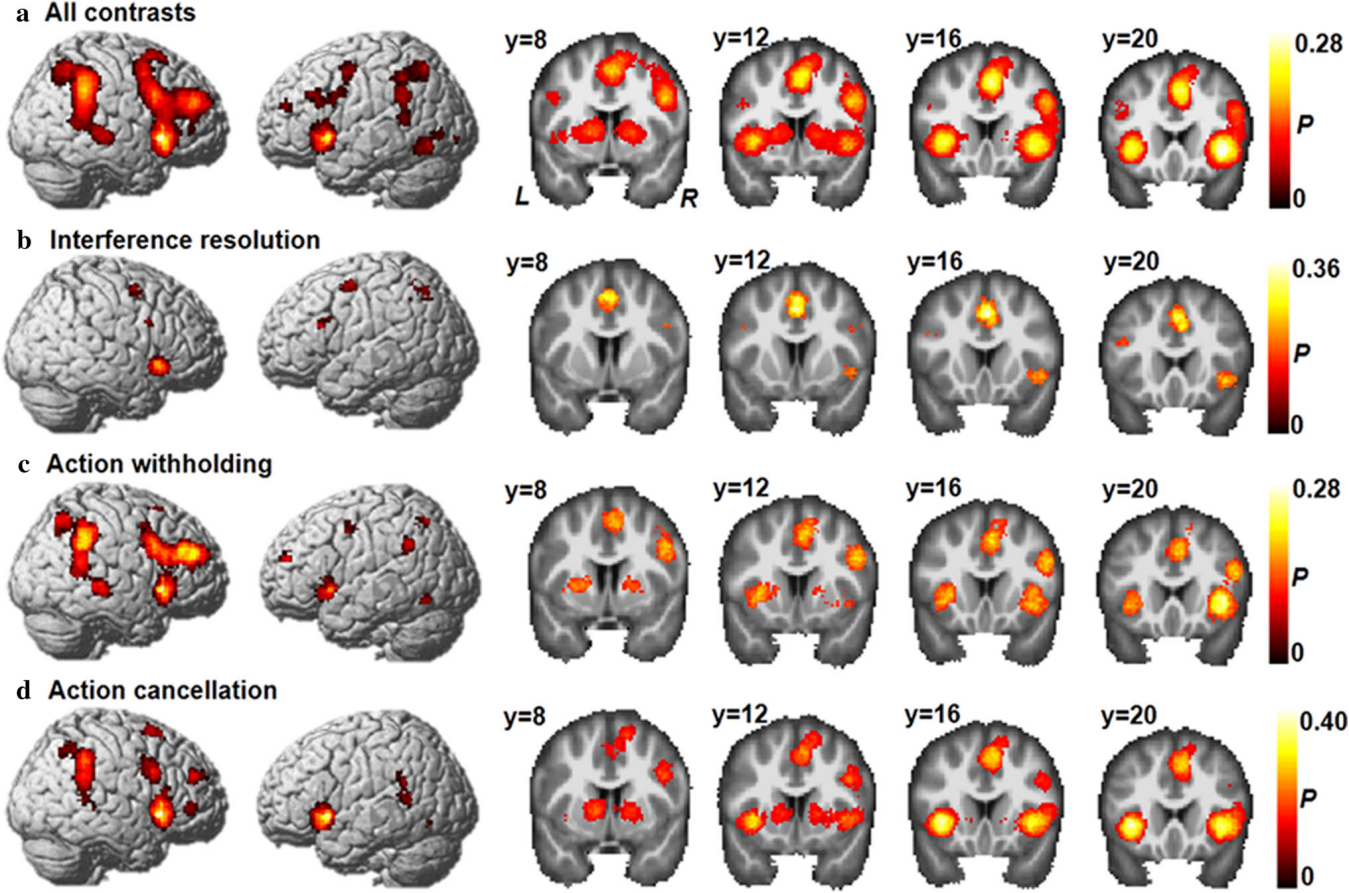
Concordance of brain activation from the MKDA analyses. **a** Brain areas activated by all contrasts. Brain areas activated in **b** interference resolution, **c** action withholding, and **d** action cancellation. The *color bar* represents the proportion of studies exhibiting the effect at the peak density weighted by sample size (*P*)

**Fig. 4:**
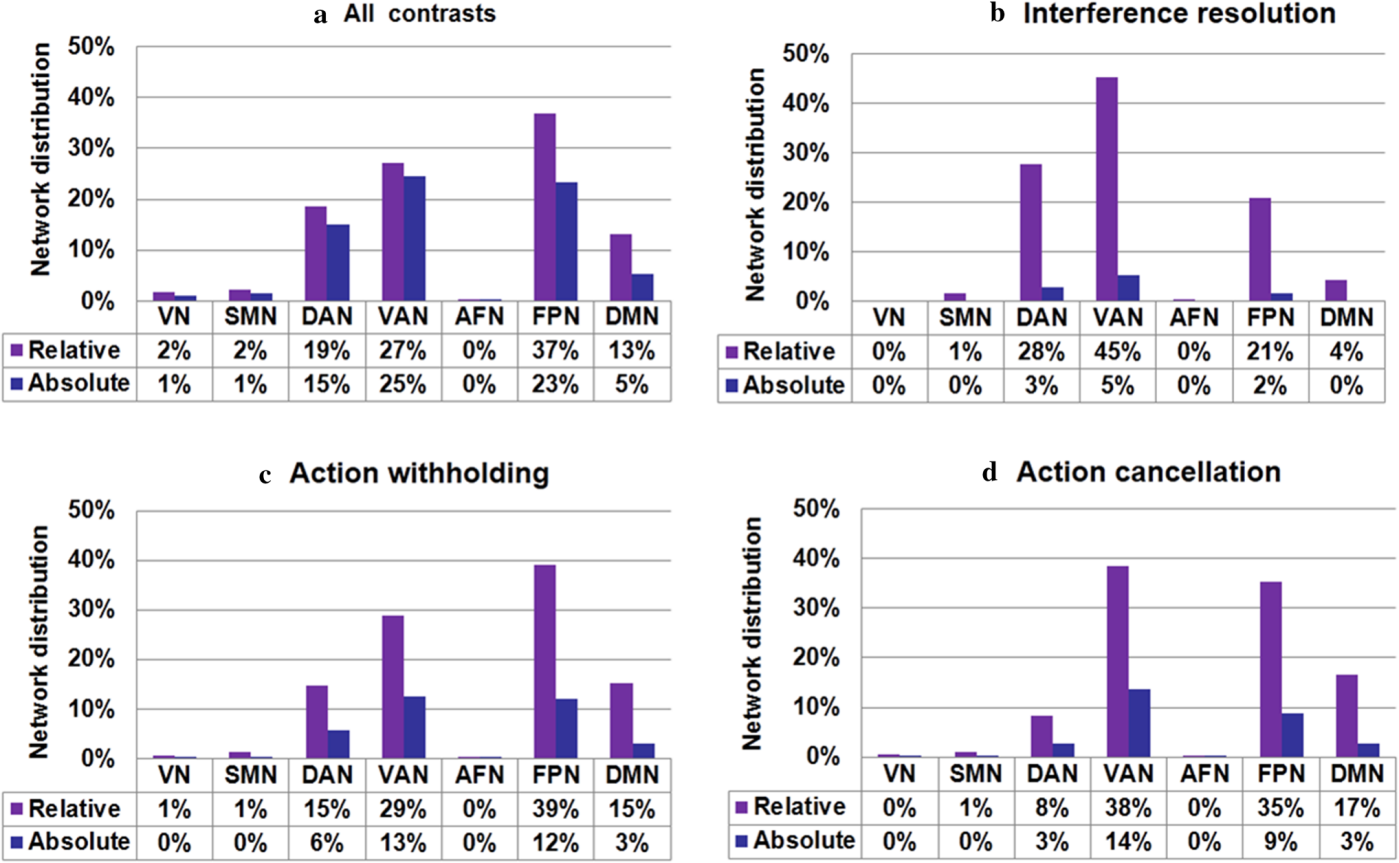
Network distribution of concordance of brain activation from the MKDA analyses. **a** Brain networks activated by all contrasts. Brain networks activated in **b** interference resolution, **c** action withholding, and **d** action cancellation. Of note, the relative distribution (relative) was estimated by the proportion of activated voxels of specific networks versus overall activated voxels; absolute distribution (absolute) was estimated by the proportion of activated voxels of specific networks versus voxels of each template network. *FPN* fronto-parietal network; *DAN* dorsal attention network; *VAN* ventral attention network; *SMN* somatomotor network; *VN* visual network; *AFN* affective network; *DMN* default mode network

The all-inclusive analysis of the 225 studies showed significant activations of three large clusters in the right hemisphere: the frontal cortex, the angular gyrus, and the supplementary motor area (Fig. 3a and Table S3 in Supplementary Materials). More precisely, cluster activations in the frontal cortex included the insula, inferior frontal gyrus, middle frontal gyrus, and superior frontal gyrus. These activations extended to the subcortical regions (the thalamus and pallidum). Furthermore, activation of the angular gyrus cluster extended to the superior temporal gyrus, while activation of the supplementary motor area extended to the middle frontal gyrus. In addition, several areas in the left hemisphere, including the insula, putamen, and middle frontal gyrus were consistently activated across all response inhibition tasks. By overlapping these clusters with the seven functional networks, we discovered that the activated areas were primarily distributed in the fronto-parietal network (36%), ventral attention network (27%), dorsal attention network (18%), and default mode network (13%) (Table S9 in Supplementary Materials) (Fig. 4a).

### Brain activation patterns of each category

#### Interference resolution

The regions activated by interference resolution were located in a subset of the previously mentioned clusters (Fig. 3b), including the left supplementary motor area, the left inferior parietal lobule, the left precentral gyrus, the right insula, the right middle frontal gyrus, and bilateral inferior frontal gyri. Based on the reported characterizations of functional neural networks, these clusters appeared to be located at the ventral attention network (45%), the dorsal attention network (27%), and the fronto-parietal network (20%) (Table 1; Fig. 4b).

**Table 1:** Brain areas significantly activated during interference resolution [*p* < 0.05, family wise error (FWE) corrected across the entire brain]

Region	R/L	MNI			Maximum *P*	No. Voxs
		*x*	*y*	*z*		
Supplementary motor area	L	0	14	46	0.36	819
Insula	R	42	18	−8	0.25	303
Inferior parietal lobule	L	−28	−58	48	0.23	229
Precentral gyrus	L	−30	−2	54	0.22	137
Middle frontal gyrus	R	32	−2	54	0.21	92
Inferior frontal gyrus, triangular part	L	−42	20	24	0.17	32
Inferior frontal gyrus, triangular part	R	46	10	28	0.19	26
Inferior frontal gyrus, triangular part	L	−46	14	28	0.18	10

Maximum *P* is the maximum proportion of studies exhibiting the effect at the peak density weighted by sample size. The coordinates are Montreal Neurological Institute (MNI) standard stereotaxic spaces. The voxel size is 2 × 2×2 mm^3^

*R/L* right/left hemisphere

#### Action withholding

There were activations in the (1) right triangular part of the inferior frontal gyrus, which extended to the insula and middle frontal gyrus; (2) right angular gyrus, which extended to the middle temporal gyrus and supramarginal gyrus; (3) right supplementary motor area, which extended to the median cingulate and paracingulate gyri; (4) left insula, which extended to the putamen; and (5) right pallidum (Table 2; Fig. 3c). The activations were distributed in the fronto-parietal network (39%), the ventral attention network (28%), the dorsal attention network (14%), and the default mode network (15%) (Fig. 4c).

**Table 2:** Brain areas significantly activated during action withholding (*p* < 0.05, FWE corrected across the entire brain)

Region	R/L	MNI			Maximum *P*	No. Voxs
		*x*	*y*	*z*		
Inferior frontal gyrus, triangular part	R	42	26	16	0.28	2706
Insula	R	44	20	−10	0.22	
Middle frontal gyrus	R	40	40	24	0.24	
Inferior frontal gyrus, opercular part	R	48	14	28	0.21	
Precentral gyrus	R	46	6	42	0.16	
Angular gyrus	R	48	−48	32	0.23	1765
Middle temporal gyrus	R	58	−32	−2	0.17	
Superior temporal gyrus	R	56	−48	14	0.17	
Supramarginal gyrus	R	52	−44	36	0.23	
Angular gyrus	R	32	−60	48	0.18	
Supplementary motor areas	R	4	14	50	0.21	983
Median cingulate and paracingulate gyri	R	4	20	40	0.20	
Insula	L	−30	14	0	0.21	691
Supplementary motor area	R	6	10	54	0.21	610
Superior parietal gyrus	L	−26	−60	50	0.16	177
Pallidum	R	20	8	4	0.17	160
Supramarginal gyrus	L	−58	−50	34	0.15	99
Inferior occipital gyrus	L	−40	−62	−10	0.14	99
Frontal_Mid_L	L	−32	50	22	0.15	64
Precentral gyrus	L	−44	−2	48	0.15	38
Precentral gyrus	R	36	2	48	0.13	14

The maximum *P* is the maximum proportion of studies exhibiting the effect at the peak density weighted by the sample size. The coordinates are Montreal Neurological Institute (MNI) standard stereotaxic spaces. The voxel size is 2 × 2×2 mm^3^

*R/L* right/left hemisphere

#### Action cancellation

The activated regions included the bilateral insula cortex, which extended to the basal ganglia (e.g., caudate and putamen) and inferior frontal gyrus; the right supplementary motor area, which extended to the median cingulate and paracingulate gyri; the bilateral superior temporal gyri; and the right inferior parietal lobule (Table 3; Fig. 3d). The activation distribution included the fronto-parietal network (35%), the ventral attention network (38%), the default mode network (16%), and the dorsal attention network (8%) (Fig. 4d).

**Table 3:** Brain areas significantly activated during action cancellation (*p* < 0.05, FWE corrected across the entire brain)

Region	R/L	MNI			Maximum *P*	No. Voxs
		*x*	*y*	*z*		
Insula	R	36	18	0	0.35	1695
Pallidum	R	18	8	2	0.20	
Inferior frontal gyrus, opercular part	R	50	18	6	0.27	
Supplementary motor area	R	6	18	48	0.29	1272
Median cingulate and paracingulate gyri	R	4	26	38	0.26	
Supramarginal gyrus	R	52	−46	36	0.28	1126
Superior temporal gyrus	R	60	−42	12	0.16	
Supramarginal gyrus	R	58	−42	34	0.29	
Inferior parietal lobule	R	34	−54	46	0.20	
Insula	L	−36	18	−4	0.40	923
Thalamus	R	4	−16	−2	0.20	410
Inferior frontal gyrus, opercular part	R	44	10	32	0.22	359
Middle frontal gyrus	R	32	46	28	0.17	164
Superior temporal gyrus	L	−58	−48	18	0.16	126
Middle frontal gyrus	R	46	42	2	0.16	85
Fusiform	L	−40	−64	−12	0.15	37

The maximum *P* is the maximum proportion of studies exhibiting the effect at the peak density weighted by the sample size. The coordinates are Montreal Neurological Institute (MNI) standard stereotaxic spaces. The voxel size is 2 × 2×2 mm^3^

*R/L* right/left hemisphere

### Common activation profiles among different classifications of response inhibition

**Fig. 5:**
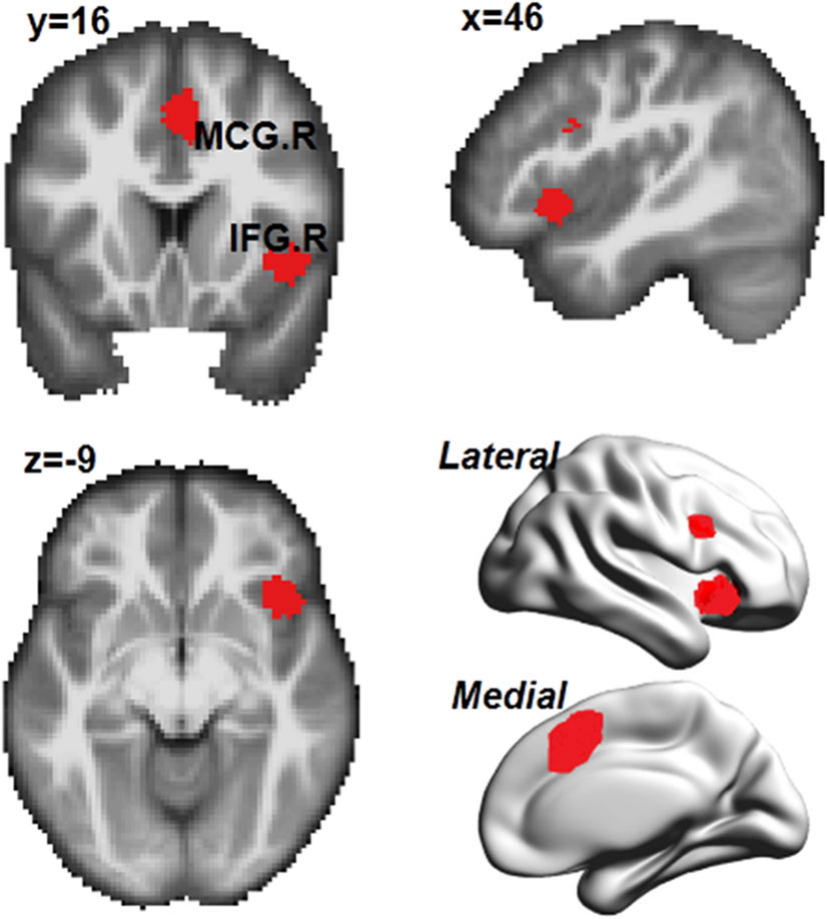
Common areas among different classifications of response inhibition. *IFG.R,* right inferior frontal gyrus, *MCG.R,* right median cingulate and paracingulate gyri

Activation patterns common to the three subcategories of processes of response inhibition were derived by overlapping their MKDA maps. The common regions included the right inferior frontal gyrus, which extended to the insula, right median cingulate, and paracingulate gyri, and the right superior parietal gyrus (Fig. 5). The network analysis showed that the common activated areas were mostly distributed in the ventral attention network (61%), fronto-parietal network (26%), default mode network (8%), and dorsal attention network (4%).

### Activation differences among different classifications

**Fig. 6:**
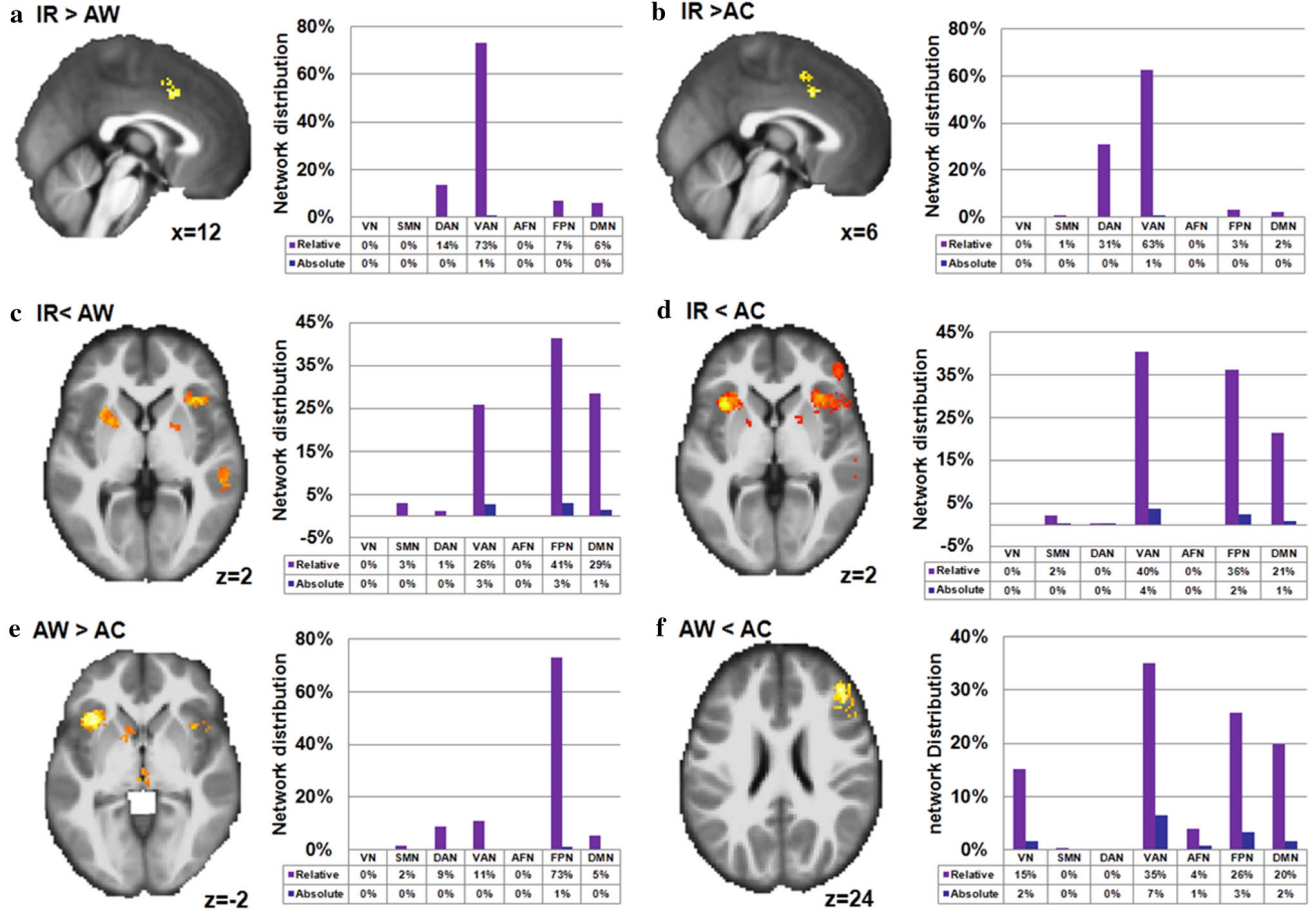
Direct contrasts of brain activations among the different classifications of response inhibition. **a** and **c** Different regions (neural network correlates) of interference resolution (IR) contrasted with action withholding (AW). **b** and **d** Different regions (neural network correlates) for interference resolution (IR) contrasted with action cancellation (AC). **e** and **f** Different regions (neural network correlates) for action withholding (AW) in contrast with action cancellation (AC). Regions showing differences between each category were listed in the *left panel*, and the corresponding neural network correlates of regions which showed differences were arranged in the *right panel*. *FPN* fronto-parietal network; *DAN* dorsal attention network; *VAN* ventral attention network; *SMN* somatomotor network; *VN* visual network; *AFN* affective network; *DMN* default mode network

Chi-square analyses revealed that the network distributions of interference resolution versus action withholding/action cancellation were significantly different (interference resolution vs. action withholding, *χ* ^2^ = 19.93, *df* = 6, *p* = 0.003; interference resolution vs. action cancellation, *χ* ^2^ = 22.37, *df* = 6, *p* = 0.001). Furthermore, we observed that interference resolution, which was related to greater activation in the ventral and dorsal attention networks (Fig. 6a, b), involved the left supplementary area, the left precentral gyrus, and the left superior parietal gyrus. On the other hand, action withholding engaged the fronto-parietal network, including regions of the right middle frontal gyrus, bilateral insula, the right triangular part of the inferior frontal gyrus, and subcortical areas such as the left putamen and right pallidum (Table 4; Fig. 6c, d).

**Table 4:** Brain activation differences among interference resolution, action withholding, and action cancellation (*p* < 0.05, FWE corrected across the entire brain)

Region	R/L	MNI			Maximum *P*	No. Voxs
		*x*	*y*	*z*		
*Interference resolution > action withholding*						
Supplementary motor area	L	−2	12	48	0.19	105
Precentral gyrus	L	−34	−2	56	0.15	10
Interference resolution < action withholding						
Middle frontal gyrus	R	40	38	26	0.21	618
Inferior frontal gyrus, triangular part	R	48	24	20	0.15	66
Inferior frontal gyrus, triangular part	R	42	34	26	0.21	308
Middle frontal gyrus	R	32	44	26	0.17	244
Superior temporal gyrus	R	56	−42	12	0.16	550
Middle temporal gyrus	R	56	−30	−2	0.15	
Supramarginal gyrus	R	56	−46	34	0.16	
Putamen	L	−28	12	2	0.15	195
Insula	R	36	22	4	0.17	115
Supramarginal gyrus	L	−58	−50	30	0.14	70
Pallidum	R	20	4	4	0.14	22
Middle frontal gyrus	L	−32	50	24	0.12	18
*Interference resolution > action cancellation*						
Supplementary motor area	L	−2	12	46	0.21	121
Superior parietal gyrus	L	−28	−54	50	0.17	18
Precentral gyrus	L	−34	−2	56	0.17	15
Interference resolution < action cancellation						
Insula	L	−34	18	−4	0.25	596
Insula	R	40	20	2	0.16	332
Inferior frontal gyrus, triangular part	R	48	20	2	0.14	
Supramarginal gyrus	R	58	−44	38	0.17	162
Supplementary motor area	R	14	14	62	0.13	74
Middle frontal gyrus	R	32	46	28	0.12	69
Middle temporal gyrus	R	58	−42	8	0.12	55
Supramarginal gyrus	L	−60	−48	24	0.13	32
Supplementary motor area	R	8	18	52	0.15	26
Superior temporal gyrus	R	56	−22	−2	0.11	26
Pallidum	R	20	4	0	0.11	22
Pallidum	L	−18	4	0	0.11	18
Middle temporal gyrus	L	−58	−52	8	0.12	14
*Action withholding > action cancelation*						
Insula	L	−36	20	−4	0.17	288
Supplementary motor area	R	4	22	52	0.12	51
Insula	R	44	16	0	0.12	43
Caudate	L	−10	10	−2	0.11	36
Action withholding < action cancelation						
Inferior frontal gyrus, triangular part	R	44	36	24	0.16	193
Middle frontal gyrus	R	42	38	24	0.16	
Supplementary motor area	R	4	2	60	0.12	25
Superior parietal gyrus	R	30	−64	54	0.12	19

The maximum *P* is the maximum proportion of studies exhibiting the effect at the peak density weighted by sample size. The coordinates are Montreal Neurological Institute (MNI) standard stereotaxic spaces. The voxel size is 2 × 2×2 mm^3^

*R/L* right/left hemisphere

Action cancellation compared with action withholding revealed a significant activation in the ventral attention and fronto-parietal networks, in which the primary correlates were the right inferior frontal gyrus, the right supplementary motor area, and the right superior parietal gyrus (Fig. 6f). Brain areas in bilateral insula and the right caudate showed greater activation during action withholding than action cancellation (Fig. 6e).

### Validation analysis

The results obtained in this study were proven replicable under different validation schemes. (1) Using the leave-one-out cross-validation procedure, we tested the effects of excluding paradigms on interference resolution and found that the neural networks of interference resolution were primarily distributed in the ventral attention network and the dorsal attention network regardless of which paradigm was excluded (Table 5: Table S9 and Fig. S1 in Supplementary). (2) When examining the NoGo vs. Go contrast in relation to action withholding, activation of the correlates of the ventral attention network and the fronto-parietal network was observed (detailed brain areas are listed in Table S7 in Supplementary Materials). (3) With regard to the contrast condition of the stop signal tasks, we found no significant differences in the distribution of neural networks, including all studies and Stop versus Go contrasts (Table 4 and Table S8 in Supplementary Materials). (4) The evaluation of the number of experiments when contrasting the three subcategories indicated no significant differences among the real contrasts and the randomly selected 68 contrasts for action withholding and action cancellation (Table 4). The activated brain areas and network distributions are displayed in Tables S4, S5, and S9 in the Supplementary Materials.

**Table 5:** Different validation schemes to test the robustness of the network correlate distribution of three kinds of response regulation using the Chi-square test

Validation protocol	*χ* ^2^	*df*	*p*
Interference resolution			
Real IR vs. IR without Flanker contrast	1.12	6	0.98
Real IR vs. IR without Simon contrasts	2.15	6	0.91
Real IR vs. IR without SRC contrasts	0.07	6	0.99
Real IR vs. IR without Stroop contrasts	7.70	6	0.26
Real IR vs. IR without WSCT contrasts	1.17	6	0.98
Real IR vs. IR without Antisaccade contrast	5.69	6	0.46
Action withholding			
Real AW vs. NoGo–Go contrasts only	0.55	6	0.99
Real AW vs. low frequency NoGo contrasts only	0.82	6	0.99
Real AW vs. high frequency NoGo contrasts only	43.52	6	<0.01
Action cancellation			
Real AC vs. Stop–Go contrast only	0.35	6	0.99
Random select contrasts			
Real All studies vs. random select all studies	1.06	6	0.98
Real AW vs. random select AW contrasts	7.02	6	0.32
Real AC vs. random select AC contrasts	1.19	6	0.97

*SRC* stimulus response compatibility; *WSCT* Wisconsin Card Sorting Test; *df* degree of freedom; *IR* interference resolution; *AW* action withholding; *AC* action cancellation

## Discussion

Through a coordinate-based meta-analysis, MKDA, we examined the neural correlates of response inhibition in different paradigms that all require the suppression of an inappropriate action and the concurrent initiation and execution of the context-appropriate alternative from a large-scale neural network perspective. Independent of the task type, brain areas including the right inferior frontal gyrus extending to the insula, the right median cingulate, and paracingulate gyri, and the right superior parietal gyrus were activated across all paradigm classes. This observation is in line with the finding of previous meta-analytic studies on the topic (e.g., Cieslik et al. 2015). By mapping the activated patterns onto the functional network atlas (Yeo et al. 2011; Choi et al. 2012), we found that the fronto-parietal network and the ventral attention network were the core neural systems engaged during response inhibition. Contrast analyses aiming to elucidate the unique neural substrates for each subcategory revealed that interference resolution, relative to action withholding/cancellation, produced stronger activation in the ventral attention network (the left supplementary area, precentral gyrus, and superior parietal gyrus). Furthermore, relative to action cancellation, action withholding primarily recruited the fronto-parietal network. On the other hand, relative to action withholding, action cancellation activated both the ventral attention and fronto-parietal networks. Overall, our results indicate common and unique neural activation patterns for the three subcategories of processes associated with response inhibition.

### Common neural networks

The fronto-parietal network is characterized as a set of cortical areas that are mutually activated when performing a wide variety of cognitively demanding tasks (Fedorenko et al. 2013; Duncan 2010). In this study, we found that across all 225 studies with 323 experiments, areas in the dorsal lateral frontal cortex, pre-supplementary motor area, and temporal parietal junction were consistently activated during all response inhibition tasks (Fig. 3a), i.e., activation was mainly distributed in the fronto-parietal network. Notably, similar results were also detected when input contrast counts were taken into account (Tables S5, S9), which demonstrated that the neural network involved in response inhibition was stable and robust. These findings are in line with the findings of previous studies on the neural correlates of working memory, sustained attention, and reasoning. Nee et al. (2013) reported that there was widespread bilateral fronto-parietal network activation during various types of working memory tasks. Similarly, consistent involvement in a very similar network was also observed in a study of the neural correlates of sustained attention (Langner and Eickhoff 2013). By synthesizing semantic and visuospatial analogy tasks, Hobeika et al. (2016) observed that there were domain-oriented regions in the inferior and middle frontal gyri. Thus, domain-oriented regions within the fronto-parietal network are widely involved in cognitive control components including response inhibition, working memory, sustained attention, and reasoning (Miyake et al. 2000; Chan et al. 2008).

By overlapping the neural activity pattern of each category of response inhibition tasks, three main clusters of activation were observed at the (1) right IFG extending to insula, (2) right median cingulate and paracingulate gyri, and (3) right superior parietal gyrus (Fig. 5). The right IFG, besides detecting changes in stimulus features (Sharp et al. 2010; Dodds et al. 2010), facilitates infrequent action-related events by activating nondominant but relevant responses while inhibiting automatic but irrelevant actions at the same time. Along this line of thought, lesion studies have also indicated that stop signal task performance worsens as the size of an inferior frontal gyrus lesion increases (Aron et al. 2003), which supports an inhibitory role of the inferior frontal gyrus in resolving conflicts during response execution. The right anterior insula has been proposed to represent a hub that controls brain activity across different tasks and stimulus modalities to initiate and adjust cognitive control mechanisms (Cai et al. 2014). The previous research has demonstrated a linear relationship between the neural activity of the anterior insula and task performance across three response inhibition tasks (Flanker, Go/NoGo etc.) (Wager et al. 2005), which were included in the current meta-analysis. With respect to the right median cingulate and paracingulate gyri (MCG), studies have revealed that the MCG is the key region for proactive rather than reactive action control, indicated by increased neural activity for endogenous action selection (Aron 2011). Notably, higher MCG activity was observed when subjects were required to perform a dual task, e.g., deciding which hand will perform the action and when to give the response (Hoffstaedter et al. 2013, 2014). Moreover, the MCG has also been implicated in performance monitoring via conflict detection in information processing, reallocation of attentional resources according to task-relevant information, and corresponding action formation (Badzakova-Trajkov et al. 2009). Friedman and Miyake 2004 indicated that subjects might experience several different mental processes during response inhibition tasks: maintaining the stimulus, which requires appropriate response in working memory; detecting conflicts for stimuli inconsistent with the goal; overcoming preponderant tendencies; and choosing correct responses.

Based on the previous findings and the current results on the common areas involved in different response inhibition tasks, we argue that when performing these tasks, individuals need to maintain task requirements across trials and goal-corresponding action inhibition through engagement of the right IFG. In contrast, the right insula is required to overcome preponderant tendencies, notice the salient stimuli, and coordinate various control mechanisms. Finally, the right MCG is engaged in monitoring conflicts so as to promote task-relevant actions. Overall, we propose that the right inferior frontal gyrus, insula, and MCG may comprise the core neural network of the supervisory attentional control system needed to implement a nondominant, context-dependent behavior against a competing behavioral alternative (Alexander and Brown 2010; Cieslik et al. 2015).

### Distinct neural networks

Our findings show that distinct neural correlates and hence networks were indicated for each of the three subcategories of processes: inference resolution, action withholding, and action cancellation. Interference resolution was found to draw on the ventral attention network, while fronto-parietal network was implicated in action withholding/cancellation.

In this meta-analysis, the experimental paradigms classified as evoking interference resolution were those that required conflict resolution and inhibition of response tendencies for successful responding (Nee et al. 2007). These paradigms induce an automatic attention reorientation and response preparation in the direction of the dominant but task-irrelevant stimuli. Participants then need to actively reorient attention to the nondominant spatial location to initiate an adequate response. This process may require enhanced consideration of the objective and engagement of resources for conflict management. Thus, inference resolution, relative to action inhibition, may draw upon significant coherent activation in the ventral attention network, especially the left pre-supplementary motor area (pre-SMA) and the left superior parietal gyrus. Pre-SMA is associated with monitoring and selecting appropriate motor response output (Nachev et al. 2008; Iannaccone et al. 2015) and the superior parietal gyrus has an essential role in re-directing attention by promoting attention allocation to nonspatial properties of stimuli (Mevorach et al. 2009; Wang et al. 2015). Overall, stronger ventral attention network activation during interference resolution compared to action inhibition may indicate that the former is to a greater extent dependent on response selection processes modulated by goals and conflicts (Nee et al. 2007).

Action withholding encompasses future action selection and inhibition, whereas action cancellation goes beyond this to demand inhibition of an ongoing response. This is induced by presenting Go and NoGo signals at the same time point in their respective trials for withholding or presenting stop signals with a delay after a Go signal for cancellation of an already initiated response. The inhibitory load is likely to be higher in cancellation than withholding (Schachar et al. 2007). Studies have suggested that the time difference in presenting the NoGo and stop signals induces distinct activation patterns (Swick et al. 2011; Rubia et al. 2001), such as a greater extent of activation in the right inferior and superior frontal gyri. This notion is partially supported by the results of our subtraction analysis between action withholding and action cancellation (Table 4). The activation differences observed between these two processes suggest that action withholding and cancellation may interact at different times within the action generation or action inhibition process, thus jointly influencing motor response inhibition (Sebastian et al. 2013a; Dambacher et al. 2014; Cieslik et al. 2015).

Further supporting the notion of time sequence difference between action withholding and action cancellation, Sebastian et al. (2013b) used a hybrid response inhibition task to study functional and spatial segregation and the specialization of underlying neural sub-processes of response inhibition and found that neural activity levels in the fronto-parietal network follow a quantitative progression: action cancellation > action withholding > interference resolution. Three stages of general information processing exist: stimulus identification, response selection, and response execution, or the motor stage. The neural resource required increased gradually when progressing through these three stages of general information processing (Schank 2014; Marois and Ivanoff 2005). Herein, the progressive increase of neural activations by interference resolution, action withholding, and action cancellation may imply that response inhibition may contain subcomponents that interact in a sequential fashion; action withholding is an intermediate process within the sequence of interference resolution, action withholding, and action cancellation (Sebastian et al. 2013a, b).

Notably, action inhibition compared to interference resolution also engages neural networks in the striatum areas, such as the putamen and pallidum. The role of subcortical areas in response inhibition has extensively been discussed (see reviews: Aron 2011; Deffains et al. 2016; Aron et al. 2016). Lesion studies also suggest that lesions of the medial striatum in rodents lead to overall longer stop signal reaction time (Eagle and Robbins 2003). In line with these animal findings, patients with damaged basal ganglia are slower to stop their responses than controls (Rieger et al. 2003). Furthermore, Go/NoGo tasks also elicit striatal activation. Many functional and structural MRI studies have pointed to a fronto-striatal “circuit” underlying response inhibition in the Go/NoGo paradigm (Durston et al. 2003; Wessa et al. 2007). In a neurophysiological experiment, striatal activity was recorded, while monkeys were performing a Go/NoGo task. The authors found that the striatum could be important for preparing to stop a response (under working memory) and then implementing inhibitory control over a sustained period (Apicella et al. 1992). Thus, the neural activity pattern in the striatum plus the frontal areas comprises a cortical circuit related to the control of response inhibition, specifically response execution (Aron 2011).

### Implications

#### General implication

In the time of cognitive neuroscience 2.0, ongoing effort is being made to develop a comprehensive cognitive atlas that defines a set of mental constructs along with a set of mental tasks and the measurement relations between those classes (Yarkoni et al. 2010; Poldrack et al. 2011). Along these lines, one effort is an increased focus on formal synthesis of the cognitive neuroscience literature using meta-analyses to establish commonalities and dissociations across tasks (Yarkoni et al. 2010). Examining whether different tasks engage shared or distinct neural correlates enhances our knowledge of the assumptions of mapping neural and mental activity and further advances our understanding of the ontology.

Several meta-analyses have contributed a lot in advancing our knowledge of response inhibition and its neural correlates (Simmonds et al. 2008; Swick et al. 2011; Criaud and Boulinguez 2013; Nee et al. 2007; Buchsbaum et al. 2005; Cieslik et al. 2015). These studies are limited, however, in that they focus on a single subcategory of response inhibition (e.g., action withholding using the Go/NoGo task only in Simmonds et al. 2008), included a small sample size (Criaud and Boulinguez 2013; Simmonds et al. 2008; Buchsbaum et al. 2005), failed to compare subcategories directly (Nee et al. 2007), or used a less reliable version of GingerALE (Eickhoff et al. 2016a). Simmonds et al. (2008), for example, included only 11 studies in their meta-analysis. It has been suggested that the replicability of meta-analyses including less than 30 studies is limited, and the conclusions drawn may, therefore, be questionable (Eickhoff et al. 2016b). Moreover, Nee et al. (2007) reported that unique neural activity patterns were associated with different response inhibitions tasks, but they did not compare these tasks to each other. Furthermore, the use of an early version of GingerALE (e.g., Swick et al. 2011; Simmonds et al. 2008), which has had reported implementation errors, may lead to false positives due to the overly liberal statistical results during multi-comparison tests (Eickhoff et al. 2016a). Our meta-analysis addresses these limitations and provides an updated quantitative meta-analytic review of the current neuroimaging literature using a dedicated updated algorithm, thus further advancing our knowledge on the neural correlates of response inhibition.

Through synthesizing data from different tasks tapping into response inhibition, we found that brain areas such as the right inferior frontal gyrus and anterior insula, which are distributed throughout the fronto-parietal network, as well as areas such as the right MCG in the ventral attentional network, are commonly activated in all of the tasks which we included. This suggests that response inhibition is one core component of cognitive control, and its neural correlates may also co-exist with other components of cognitive control (Duncan 2010). Meanwhile, subtraction analyses suggested that some specific neural correlates are involved in different categories of response inhibition tasks, which indicates that response inhibition is not a unidimensional construct. Instead, response inhibition could be a multidimensional construct consisting of multiple subcategories of cognitive processes, which recruit both common and distinct neural correlates, and hence neural networks. Sebastian et al. (2013b) conducted a well-designed study that used a hybrid response inhibition task to demonstrate that the subcomponents of response inhibition included in the current meta-analysis (i.e., interference inhibition, action withholding, and action cancellation) intervened in the action generation process at different points in time to implement response inhibition, further supporting our findings. Further methodologically rigorous studies are needed to develop and validate measurement tools specific to each subcategory of the response inhibition process.

#### Clinical implications

Regarding the core neural correlates of response inhibition, one implication is that individuals who are impaired in one kind of response inhibition task may also be impaired in other tasks, because common activations unite the different response inhibition tasks, such as within the right inferior frontal gyrus. Accordingly, individuals with motor action inhibition deficits may also demonstrate impairment in interference resolution. For example, a specific deficit in inhibiting proponent motor responses during the stop signal task (Mittner et al. 2014) as well as cognitive inhibition in the Stroop task (Lynn et al. 2014; Ganos et al. 2014) was observed in methamphetamine abusers. Revealing the core neural correlates of response inhibition also provides new insights into cognitive training. Establishing specific training programs for specific components of response inhibition is generally difficult, and the incorporation of all aspects of the cognitive process into the training program is impractical. However, enhanced performance in one or two response inhibition tasks may improve the efficiency of the right inferior frontal gyrus-based inhibition process and produce long-term benefits that affect regulation across multiple response inhibition contexts. Thus, individuals may be able to begin with more manageable tasks and progress to the tasks that target the cognitive functions that contribute to dysfunctional inhibition in their daily lives. Moreover, specific neural correlates for each subcategory of response inhibition may also help us to identify phenotypes for mental disorders (Van Belle et al. 2014), such as schizophrenia and ADHD. Schachar et al. (2007) conducted a successful trial using variations of the stop signal task to evaluate the convergence of action withholding and action cancellation and to determine whether ADHD was marked by a deficit in one or both of these executive control processes. Both action withholding and cancellation are impaired in ADHD individuals. Similar studies have begun to emerge (Johnstone et al. 2009), and precise knowledge of the distinct profiles of response inhibition subcomponents will advance diagnostic accuracy.

### Methodological considerations

We acknowledge that our meta-analysis is subject to limitations. The first one is related to the bias of synthesizing different tasks into the components of interference resolution. Our meta-analysis included studies adopting a mixture of inhibitory control tasks, such as the Stroop, Simon, and Flanker tasks, with the consequence of increased heterogeneity of study paradigm and designs. We adopted the leave-one-out cross-validation method to test homogeneity and determined that all brain activation areas showed highly replicable network distribution profiles (Table S9; Figure S1 in Supplementary Materials). Nevertheless, future meta-analyses including additional studies with common stimulus–response incompatibility tasks are needed. The second limitation is related to the use of the Go/NoGo task for action withholding. Go/NoGo can be classified as a simple task (the NoGo stimulus was always the same) or a complex task (the NoGo stimulus changed depending on context) that may require more frequent updating of stimulus–response association in working memory (Simmonds et al. 2008). As such, simple and complex Go/NoGo tasks may not be perfectly matched across the included studies. In line with this, it has been suggested that the activity pattern in the fronto-parietal network may be derived from the high demand placed on attentional or working memory resources (Simmonds et al. 2008; Criaud and Boulinguez 2013). However, such suggestions were derived from meta-analyses that included less than 30 experiments and thus may not be sufficiently robust (Eickhoff et al. 2016b). We compared high (simple) and low (complex) frequencies of NoGo stimulus (i.e., equal to 50% and less than 50%, respectively) and found that the network correlate distributions of activated areas did not differ significantly (Tables S6, S7, respectively, in the Supplementary Materials). Third, the use of the data generated from stop signal tasks to assess action cancellation drew more on proactive than reactive control for inhibiting inappropriate responses (Aron 2011; Vink et al. 2014; Van Belle et al. 2014; Vink et al. 2015). To control for this confounding effect, we have conducted a supplementary Chi-square test. The result of this test demonstrated that the network correlate distributions between all contrasts and those contrasts that included only the Stop–Go tasks did not differ significantly (detailed condition comparisons of each stop signal task and the results are presented in Tables S2 and S9 in the Supplementary Materials). Nonetheless, despite the robust findings in this study, the heterogeneity of the action cancellation tasks should be a point of concern for future research.

## Conclusions

In this meta-analysis, we examined the neural basis of three subcategories of cognitive processes underpinning response inhibition, namely interference resolution, response withholding, and response cancellation. We followed a neural network perspective with multi-kernel density analysis and reviewed studies employing tasks that require inhibition of inappropriate actions as well as concurrent initiation and execution of context-appropriate alternatives. Independent of the task types, activation of the right hemispheric regions (the IFG, insula, median cingulate, and paracingulate gyri) and the superior parietal gyrus was common across the cognitive processes studied. Mapping the activation patterns to a brain functional network atlas revealed that the fronto-parietal and the ventral attention network were the core neural systems commonly engaged during the different processes of response inhibition. Subtraction analyses elucidated the distinct neural substrates of interference resolution, action withholding, and action cancellation, and revealed stronger activation in the ventral attention network for interference resolution than action inhibition. On the other hand, action withholding/cancellation primarily engaged the fronto-striatal circuit. Overall, our results suggest that response inhibition is not a unidimensional construct but consists of subcategories of cognitive processes that engage common as well as distinct neural correlates and networks. This finding has significant implications for the knowledge and assessment of response inhibition and its related clinical conditions.

## Electronic supplementary material

Below is the link to the electronic supplementary material.

Supplementary material 1 (DOC 905 kb)
