# Supplementary material for: Large-scale functional neural network correlates of response inhibition: an fMRI meta-analysis
Source: Brain Struct Funct. 2017 May 27;222(9):3973–90. doi: 10.1007/s00429-017-1443-x (PMC5686258; doi:10.1007/s00429-017-1443-x)
Supplement: Supplementary file 1 — Supplementary material 1 (DOC 905 kb) [file 429_2017_1443_MOESM1_ESM.doc]

**Supplementary Notes**

**Large-scale functional neural network correlates of response inhibition: An fMRI [meta-analysis](http://www.sciencedirect.com.eproxy1.lib.hku.hk/science/article/pii/S0149763413002261#200008332)**

Ruibin Zhang ^a,b†^, Xiujuan Geng ^a,b,c†^, Tatia M.C. Lee ^a,b,c,d^

^a^ Laboratory of Neuropsychology, The University of Hong Kong, Hong Kong.

^b^ Laboratory of Cognitive Affective Neuroscience, The University of Hong Kong, Hong Kong.

^c^ The State Key Laboratory of Brain and Cognitive Sciences, The University of Hong Kong, Hong Kong.

^d^ Institute of Clinical Neuropsychology, The University of Hong Kong, Hong Kong.

^†^ Both authors contributed equally to this work

**Correspondence to:**

Tatia M.C. Lee, Ph.D.

May Professor in Neuropsychology

Rm 656, Jockey Club Tower

The University of Hong Kong

Pokfulam Road

Hong Kong

Tel: (852) 3917-8394

Email: tmclee@hku.hk

***Running title:*** Meta-analysis of response inhibition

**List of Tables**

Table S1 Searching results with different searching keywords under PubMed and Web of Science from 2001/01/01 to 2015/12/31.

Table S2 The characteristics of the included studies.

Table S3 Brain activation patterns from all studies (*p* < 0.05, family-wise error (FWE) corrected across the entire brain).

Table S4 Brain areas activated by 68 randomly selected contrasts from action withholding and action cancellation papers (*p* < 0.05, FWE corrected across the whole brain).

Table S5 Brain areas activated by all randomly selected studies (*p* < 0.05, FWE corrected across the entire brain).

Table S6 Brain areas activated by low (<50%) vs. high (50%) frequencies of NoGo stimulus from the MKDA analysis (*p* < 0.05, FWE corrected across the entire brain).

Table S7 Brain areas activated by NoGo vs Go trials from the MKDA analysis (*p* < 0.05, FWE corrected across the entire brain).

Table S8 Brain areas activated by Stop vs Go trials only from the MKDA analysis (*p* < 0.05, FWE corrected across the entire brain).

Table S9 The distribution of brain activation patterns overlapped on the functional network template.

***Searching strategy:***

Key words like ‘fMRI and response inhibition’; ‘fMRI and inhibitory control’, ’fMRI and interference resolution’, ‘fMRI and stoping’, ’fMRI and stop signal’, ‘fMRI and go no-go’, ‘fMRI and action cancelation’, ‘fMRI and action restraint’, and ‘fMRI and countermanding’ were searched under Pubmed and Web of Science with time frame from 2001/01/01 to 2015/12/31. In addition, the reference lists included in the selected fMRI studies were checked. The detailed searching results can be seen in Table. S1.

Table S1. Searching results with different searching keywords under PubMed and Web of Science from 2001/01/01 to 2015/12/31.

| Search key words | Data Source | |
| --- | --- | --- |
|  | PubMed | Web of science |
| response inhibition & fMRI | 1370 | 1783 |
| inhibitory control & fMRI | 764 | 770 |
| interference resolution & fMRI | 42 | 156 |
| stopping & fMRI | 226 | 421 |
| stop signal & fMRI | 266 | 272 |
| go no-go & fMRI | 306 | 387 |
| action restraint & fMRI | 15 | 9 |
| action cancellation & fMRI | 9 | 11 |
| action withholding & fMRI | 11 | 17 |
| countermand & fMRI | 4 | 24 |

***Supplementary analysis***

***Effects of paradigm***

In this study, we used different paradigms for interference resolution, which may have biased the accurate detection of the neural correlates of response inhibition. Jackknife analyses were conducted to assess whether specific paradigms (e.g., Flanker) disproportionately affected the results. To accomplish this task, the density statistic for each significant cluster was iteratively recalculated with each paradigm omitted, and a χ2 test was performed on the proportion of activated voxels between the original density statistic and the leave-one-out density statistic.

***Effects of NoGo frequency***

Criaud et al. (2013) have conducted a systematic review of the variants on Go/NoGo paradigms and have suggested that the effect of the NoGo frequency should be considered during the meta-analysis. To test the robustness of our main results, we followed Criaud’s work and compared the difference between the experiments with high frequency of NoGo stimulus (50%) and the experiments with a low probability of NoGo stimulus (<50%) (73% experiments). The detailed NoGo frequency of each experiment is listed in Table S1.

***Effect of contrast condition***

For the majority of the Go/NoGo experiments (78%), NoGo trials were contrasted with Go trials, whereas the remaining contrasts compared the NoGo trials versus fixation (e.g., ) or a low-level baseline (e.g., ). Regarding the stop signal task, the most appropriate comparison conditions have been substantially debated in the literature . In this study, approximately 76 experiments (68%) contrasted Stop trials with Go trials. In addition, other studies have reported contrasts that included successful stop vs. unsuccessful stop trials and Stop vs. oddball stimulus . A separate analysis of NoGo vs. Go trials and Stop vs. Go trials was used to test the contrast condition effect on the results.

Table S2 The characteristics of included studies.

| Study (Author ) | Sample (M/F) | | Field strength | Design  (B/E) | | Paradigm | Comparison | | foci | | | Space | | |
| --- | --- | --- | --- | --- | --- | --- | --- | --- | --- | --- | --- | --- | --- | --- |
| ***Interference resolution*** | | | | | | | | | | | | | |  |
| Blasi et al | | 32/25 | 3 | E | Modified flanker, 50% incongruent trials | | | InCongruent > Congruent/Neutral | | | 11 | | TAL |  |
| Brass et al | | 8/12 | 3 | E | Stroop, 50% incongruent trials | | | InCongruent > Congruent | | | 7 | | TAL |  |
|  |  |  |  |  | Imitation-inhibition, 50% incongruent trials | | | InCongruent > Congruent | | | 8 | |  |  |
| Brass et al | | 4/6 | 3 | B | Stroop, 50% incongruent trials | | | InCongruent > Congruent | | | 5 | | TAL |  |
| Brown et al | | 4/6 | 4 | E | Antisccade, 33% antisccade trials | | | Antisccade > Base line | | | 20 | | TAL |  |
| Buge et al | | 9/7 | 3 | E | Flanker, 25% incongruent trials | | | InCongruent > Neutral (adults) | | | 11 | | TAL |  |
| Chikaze et al | | 10/12 | 1.5 | E | Antisaccade, 13.5% antisaccade trials | | | Antisaccade > Control Saccade | | | 55 | | TAL |  |
| Forstmann et al. | | 3/11 | 3 | B | Simon | | | InCongruent > Congruent | | | 1 | | MNI |  |
| Fortmann et al. | | 9/15 | 3 | B | Simon | | | InCongruent >Neutral | | | 6 | | MNI |  |
| Motoshita et al. | | 9/9 | 1.5 | B | Antisaccade, 50% antisaccade trials | | | Antisaccade > Rest (Control) | | | 12 | | MNI |  |
| Garavan et al. | | 8/6 | 1.5 | E | Response-inhibition task | | | Inhibitt > Respond | | | 14 | | TAL |  |
| Hazltine et al. | | 3/5 |  | E | Flanker, 50% incongruent trials | | | InCongruent > Congruent | | | 4 | | TAL |  |
| Kim et al. | | 8/5 | 3 | E | Stroop | | | InCongruent > Neutral | | | 7 | | MNI |  |
| Konishi et al. | | 35/43 | 1.5 | B | WCST | | | Control > Release Trials | | | 22 | | TAL |  |
| Liu et al. | | 3/8 | 1.5 | E | Simon Stroop | | | Simon InCongruent > Congruent | | | 34 | | MNI |  |
|  |  |  |  |  |  |  |  | Stroop InCongruent > Congruent | | | 15 | |  |  |
| Matsuda et al. | | 21 | 1.5 | B | Antisaccade/Saccade | | | Antisaccade > Rest | | | 17 | | MNI |  |
|  |  |  |  |  |  |  |  | Antisaccade > Saccade | | | 12 | |  |  |
| Mayer et al. | | 12/12 | 1.5 | E | Stroop | | | InCongruent > Congruent | | | 17 | | TAL |  |
| Mcdowell et al. | | 13/1 | 1.5 | B | Antisaccade/ Saccade | | | Antisaccade > Saccade | | | 1 | | TAL |  |
| McNab et al. | | 4/7 | 1.5 | E | Flanke, 50% incongruent trials | | | InCongruent > Congruent | | | 5 | | MNI |  |
| Mitchell et al. | | 4/11 | 3 | B | Stroop | | | InCongruent > Neutral | | | 23 | | TAL |  |
| Potenza et al. | | 11 | 1.5 | E | Stroop, 8% incongruent trials | | | InCongruent > Congruent | | | 10 | | TAL |  |
| Reuter et al. | | 19 | 1.5 | E | Saccade | | | Inhibition condition > Uninhibition condition | | | 7 | | MNI |  |
| Rubia et al. | | 23/0 | 1.5 | E | Simon, 12% incongruent troals | | | InCongruent > Congruent | | | 9 | | TAL |  |
| Schwerdtfeger et al. | | 14 | 3 | E | Antisaccade | | | Correct Antisaccade > Fixation (control) | | | 10 | | TAL |  |
| Sebastian et al. | | 11/13 | 3 | E | Simon, 50% incongruent trials | | | InCongruent > Congruent | | | 10 | | MNI |  |
| Sebastian et al. | | 12/9 | 3 | E | Hybrid Respo nse Inhibition task | | | InCongruent Go > Congruent Go | | | 24 | | MNI |  |
|  |  |  |  |  | Simon, 50% incongruent trials | | | InCongruent Go > Congruent Go | | | 22 | |  |  |
|  |  |  |  |  | Conjunction analysis | | | Successful inhibition > Go | | | 17 | |  |  |
| Tu et al. | | 5/5 | 3 | B | Antisaccade | | | Antisaccade > Rest condition (control) | | | 21 | | MNI |  |
| Wager et al. | | 14 | 3 | B | Flanker | | | InCongruent > Congruent | | | 9 | | TAL |  |
|  |  |  |  |  | Flanker | | | Unique | | | 5 | |  |  |
|  |  |  |  |  | Stimulus–response compatibility task | | | Opposite > Same | | | 12 | |  |  |
|  |  |  |  |  | Stimulus–response compatibility task | | | Unique | | | 5 | |  |  |
|  |  |  |  |  |  | | | Conjunction analysis | | | 5 | |  |  |
| Wagner et al. | | 0/16 | 1.5 | E | Stroop, 50% incongruent trials | | | InCongruent > Congruent (control) | | | 2 | | TAL |  |
|  | | 6/14 | 1.5 | B | Stroop | | | General negative words > Neutral words (control)  Individual negative words > Neutral words, (control) | | | 7  19 | | MNI |  |
| Konishi et al. | | 18/18 | 1.5 | E | WCST | | | Inhibition trials > Control (Exp1) | | | 16 | | TAL |  |
|  |  |  |  |  |  |  |  | Inhibition trials > Control (Exp2) | | | 21 | |  |  |
| Ford et al. | | 7/3 | 4.0 | E | Antisaccade | | | Antisaccade > Prosaccade | | | 8 | | TAl |  |
|  |  |  |  |  |  |  |  | Correct Anti > Error Anti | | | 3 | | TAL |  |
| Mead et al (2002) | | 18 | 1.5 | B | Stroop | | | Incongruent > Congruent | | | 1 | | MNI |  |
| Peterson et al (2002) | | 5/5 | 1.5 | E | Stroop | | | Incongruent > Congruent | | | 14 | | MNI |  |
| Peterson et al (2002) | | 5/5 | 1.5 | E | Simon | | | Incongruent > Congruent | | | 14 | | MNI |  |
| Erickson et al (2004) | | 5/7 | 1.5 | B | Stroop | | | Incongruent > Baseline | | | 5 | | TAL |  |
| Schmiz et al (2006) | | 12 | 1.5 | E | Stroop | | | Incongruent > Congruent | | | 5 | | TAL |  |
| Wittfoth et al (2006) | | 3/27 | 3 | E | Simon | | | Incongruent > Congruent (Motion) | | | 10 | | TAL |  |
| Wittfoth et al (2006) | | 3/27 | 3 | E | Simon | | | Incongruent > Congruent (location) | | | 11 | | TAL |  |
| Bernal et al (2009) | | 8/10 | 1.5 | E | Stroop | | | Incongruent > Congruent | | | 38 | | TAL |  |
| Page et al (2009) | | 10 | 1.5 | E | Stroop | | | Incongruent > Congruent | | | 1 | | TAL |  |
| Zhu et al (2010) | | 23 | 3 | E | Flanker | | | Incongruent >Congruent | | | 8 | | TAL |  |
| Pompei et al (2011) | | 23/25 | 1.5 | B | Stroop | | | Incongruent >Congruent | | | 9 | | MNI |  |
| Zurawska et al (2011) | | 2/22 | 3 | B | Flanker | | | Incompatible > Compatible | | | 6 | | MNI |  |
| Grandjean et al (2012) | | 12/13 | 3 | B | Stroop | | | Incongruent > Neutral | | | 19 | | MNI |  |
| King et al (2012) | | 11/14 | 3 | B | Flanker | | | Incongruent >Congruent | | | 9 | | MNI |  |
| Sebastian et al (2013) | | 49 | 3 | E | Simon | | | Incongruent >Congruent | | | 26 | | MNI |  |
| Korsch et al (2014) | | 10/10 | 3 | B | Flanker | | | Incongruent > Congruent | | | 4 | | MNI |  |
| Korsch et al (2014) | | 10/10 | 3 | B | SRC | | | Incongruent > Congruent | | | 3 | | MNI |  |
| Korsch et al (2014) | | 10/10 | 3 | B | Flanker+SRC | | | Incongruent > Congruent | | | 8 | | MNI |  |
| Berron et al (2015) | | 11/13 | 3 | B | Flanker | | | Incongruent > Congruent | | | 10 | | MNI |  |
| Iannacccone et al (2015) | | 7/8 | 3 | B | Flanker | | | Correct Incongruent > Incorrect incongruent | | | 13 | | MNI |  |
| Purmann et al (2015) | | 8/12 | 3 | B | Stroop | | | Incongruent > Congruent | | | 7 | | MNI |  |
| Song et al (2015) | | 10/10 | 3 | B | Stroop | | | Incongruent > Congruent | | | 7 | | TAL |  |
| Song et al (2015) | | 10/10 | 3 | B | Reverse Stroop | | | Incongruent > Congruent | | | 11 | | TAL |  |
| Spielberg et al (2015) | | 37/64 | 3 | B | Reverse Stroop | | | Incongruent > Congruent | | | 12 | | MNI |  |
| ***Action withholding*** | | | | | | | | | | | | | |  |
| Altshuler et al. | | 5/8 | 3 | B | Go/NoGo, 50% NoGo signals | | | NoGo > Go (control) | | 4 | | | MNI |  |
| Asahi et al. | | 10/7 | 1.5 | B | Go/NoGo, 50% NoGo signals | | | NoGo > Go. | | 11 | | | TAL |  |
| Baumeister et al | | 12/1 | 3 | E | NoGo, 23% NoGo signals | | | NoGo > Neutral condition | | 16 | | | MNI |  |
| Baglio et al (2001) | | 7/4 | 1.5 | E | Go/NoGo, 20% NoGo trials | | | NoGo- fixation, controls | | 5 | | | TAL |  |
| Behan et al (2015) | | 9/11 | 3 | E | MID-Go/NoGo, 33% NoGo signals | | | Monetary incentive delay NoGo | | 3 | | | TAL |  |
| Bellgrove et al (2004) | | 13/29 | 1.5 | E | Go/NoGo, 8% NoGo signals | | | Successful NoGo > Go, | | 19 | | | MNI |  |
| Berkman et al (2009) | | 6/8 | 3 | B | Go/NoGo, 20% NoGo signals | | | NoGo >Go, | | 6 | | | MNI |  |
|  |  |  |  |  |  |  |  | Negative NoGo >Go | | 10 | | |  |  |
|  |  |  |  |  |  |  |  | Positive NoGo >Go | | 3 | | |  |  |
| Blasi et al (2006) | | 32/25 | 3 | E | Modified flanker, 50% incongruent trials | | | NoGo >Congruent/ Neutral | | 16 | | | TAL |  |
|  |  |  |  |  |  |  |  | Incongrent > Congruent/ Neutral | | 11 | | |  |  |
|  |  |  |  |  |  |  |  | Response inhibition > Interference control | | 13 | | |  |  |
|  |  |  |  |  |  |  |  | Response inhibition < Interference control | | 1 | | |  |  |
| Borgward et al (2008) | | 25/0 | 1.5 | E | Go/NoGo, 11% NoGo trials | | | No-Go > Oddball Trials, placebo | | 5 | | | TAL |  |
| Braver et al (2001) | | 5/9 | 1.5 | E | Go/NoGo, 17% NoGo trials | | | NoGo > Go | | 11 | | | TAL |  |
| Brosch et al (2011) | | 8/11 | 3 | E | Go/NoGo, 10% NoGo trials | | | NoGo correct > NoGo error | |  | | | MNI |  |
| Brown et al (2006) | | 4/6 | 4 | E | NoGo, 33% NoGo trials | | | NoGo > Base line | | 19 | | | TAL |  |
| Brown et al (2012) | | 7/13 | 4.7 | E | Go/NoGo, 20% NoGo trials | | | NoGo > Go | | 17 | | | MNI |  |
| Brown et al (2008) | | 6/5 | 4 | E | Saccade and NoGo, 33% NoGo trials | | | Rare NoGo > Frequent saccades | | 8 | | | TAL |  |
| Brown et al (2006) | | 21/37 | 3 | B | Go/NoGo, 25% NoGo trials | | | NoGo > Go | | 5 | | | MNI |  |
| Buge et al (2002) | | 9/7 | 3 | E | NoGo, 25% NoGo trials | | | NoGo > Neutral (adults) | | 16 | | | TAL |  |
| Burke et al (2011) | | 5/6 | 3 | Mixed | Go/NoGo, 25% NoGo trials | | | NoGo> Rand condition | | 11 | | | MNI |  |
|  |  |  |  |  |  |  |  | NoGo > Go (First/Second presentation) | | 17 | | |  |  |
| Chen et al (2014) | | 15/0 | 3 | B | Go/NoGo, 50% NoGo trials | | | NoGo > Go | | 7 | | | MNI |  |
| Chiang et al (2013) | | 6/10 | 3 | E | Go/NoGo, 20% NoGo trials | | | NoGo > Go | | 9 | | | TAL |  |
| Chikazoe et al (2009) | | 10/15 | 1.5 | E | Go/NoGo, 12.3% NoGo trials | | | NoGo > Frequent Go | | 52 | | | TAL |  |
|  |  |  |  |  |  |  |  | NoGo > Infrequent Go | | 52 | | |  |  |
| Chuah et al (2006) | | 15/12 | 3 | Mixed | Go/NoGo, 10% NoGo trials | | | Success NoGo > Go (E) | | 5 | | | TAL |  |
|  |  |  |  |  |  |  |  | Blocked task effects > fixation (B) | | 9 | | |  |  |
| de Zubicary et al (2000) | | 8/0 | 1.5 | B | Go/NoGo, 26% NoGo trials | | | Increased activations for refrain vs Go | | 15 | | | TAL |  |
|  |  |  |  |  |  |  |  | Linear increases with trials equated per block | | 11 | | | TAL |  |
| Dillo et al (2010) | | 11/4 | 1.5 | B | Go/NoGo, 50% NoGo trials | | | NoGo > Go (controls) | | 2 | | | TAL |  |
| Dodds et al (2011) | | 13/7 | 3 | B | Go/NoGo, 11% NoGo trials | | | NoGo > Go | | 3 | | | MNI |  |
| Duerden et al (2013) | | 15/5 | 3 | B | Go/NoGo, 20% NoGo trials | | | NoGo > Go (Controls) | | 13 | | | MNI |  |
| Durston et al (2000) | | 5/5 | 1.5 | E | Go/NoGo, 25% NoGo trials | | | NoGo > Go | | 10 | | | TAL |  |
| Falconer et al (2008) | | 10/13 | 1.5 | E | Go/NoGo, 25% NoGo trials | | | NoGo > Go (Control) | | 6 | | | MNI |  |
| Fassbender et al (2004) | | 7/14 | 1.5 | Mixed | Go/NoGo, 10% NoGo trials | | | Blocked task effects > Rest | | 21 | | | TAL |  |
|  |  |  |  |  |  |  |  | NoGo > Go, Correct | | 8 | | |  |  |
| Fassbender et al (2009) | | 5/10 | 1.5 | E | Go/NoGo, 7% NoGo trials | | | Correct NoGo > Go | | 7 | | | TAL |  |
| Fassbender et al (2006) | | 6/11 | 1.5 | B | Go/NoGo, 50% NoGo trials | | | Correct NoGo > Go | | 18 | | | TAL |  |
| Fedota et al (2014) | | 9/7 | 3 | E | Go/NoGo, 17% NoGo trials | | | NoGo > Go | | 9 | | | MNI |  |
| Garavan et al (2006) | | 25/46 | 1.5 | E | Go/NoGo, 5% NoGo trials | | | Successful NoGo > Go | | 20 | | | TAL |  |
| Garavan et al (2002) | | 4/10 | 1.5 | E | Go/NoGo, 6% NoGo trials | | | Successful NoGo > Go | | 16 | | | TAL |  |
| Garavan et al (2003) | | 6/10 | 1.5 | B | Go/NoGo, 10% NoGo trials | | | Block task effects > Rest | | 12 | | | TAL |  |
|  |  |  |  |  |  |  |  | Successful NoGo > Go | | 7 | | |  |  |
| Goghari et al (2009) | | 7/5 | 3 | E | Go/NoGo, 20% NoGo trilas | | | NoGo >Go, Probe-related activity | | 8 | | | MNI |  |
| Goldstein et al (2007) | | 4/10 | 3 | B | Go/NoGo, 37.5% NoGo trials | | | Negative NoGo > Go | | 28 | | | MNI |  |
|  |  |  |  |  |  |  |  | Positive NoGo > Go | | 1 | | |  |  |
| Maldonado et al (2010) | | 10/11 | 3 | B | Go/NoGo, 20% NoGo trials | | | NoGo>Go | | 2 | | | TAL |  |
| Mobbs et al (2005) | | 2/9 | 1.5 | B | Go/NoGo | | | Go/NoGo blocks > Go | | 4 | | | TAL |  |
| Hare et al (2005) | | 5/5 | 3 | B | Go/NoGo, 30% NoGo trials | | | NoGo > Go | | 3 | | | TAL |  |
| Hester et al (2004) | | 7/8 | 1.5 | E | Go/NoGo, 12% NoGo trials | | | Successful NoGo > Go | | 33 | | | TAL |  |
| Hester et al (2004) | | 5/10 | 1.5 | Mixed | Cue Go/NoGo, 6% NoGo trials | | | Successful NoGo>Go | | 21 | | | TAL |  |
| Holz et al (2014) | | 182 | 3 | E | Go/NoGo, 23% NoGo trials | | | NoGo> Neutral | | 23 | | | MNI |  |
| Horn et al (2003) | | 21/0 | 1.5 | B | Go/NoGo, 25% NoGo trials | | | NoGo > Go | | 14 | | | TAL |  |
| Kiehl et al (2000) | | 7/7 | 1.5 | E | Go/NoGo, 20% NoGo trials | | | NoGoNGo, correct rejects | | 8 | | | TAL |  |
| Jacob et al (2013) | | 18 | 3 | Mix | Go/NoGo, 37.5% NoGo trials | | | NoGo > Go (control) | | 3 | | | MNI |  |
| Jamadar et al (2010) | | 7/11 | 1.5 | B | Go/NoGo, 33% NoGo trials | | | NoGo > Informatively cued (Go) | | 43 | | | TAL |  |
| Kaladjian et al (2007) | | 19/2 | 3 | E | Go/NoGo, 50% NoGo trials | | | Correct NoGo > Correct Go (control) | | 11 | | | TAL |  |
| Kaladjian et al (2009) | | 5/5 | 3 | E | Go/NoGo, 50% NoGo trials | | | Correct NoGo > Correct Go (T1 Control) | | 12 | | | TAL |  |
|  |  |  |  |  |  |  |  | Correct NoGo > Correct Go (T2 Control) | | 8 | | |  |  |
| Kaladjian et al (2009) | | 5/5 | 3 | E | Go/NoGo, 50% NoGo trials | | | Correct NoGo > Correct Go (control) | | 16 | | | TAL |  |
| Karch et al (2008) | | 14 | 1.5 | E | Go/NoGo, 50% NoGo trials | | | NoGo > Control condition (control) | | 13 | | | TAL |  |
| Kelly et al (2004) | | 5/10 | 1.5 | E | Go/NoGo, 8% NoGo trials | | | Successful NoGo > Go, slow and fast | | 23 | | | TAL |  |
|  |  |  |  |  |  |  |  | Successful NoGo > Go, Fast>Slow | | 7 | | |  |  |
| Ko et al (2014) | | 23/0 | 3 | E | Go/NoGo, 17% NoGo trials | | | Successful NoGo > Go (control) | | 1 | | | MNI |  |
| Konishi et al (1999) | | 5/1 | 1.5 | E | Go/NoGo, 50% NoGo trials | | | NoGo > Go, NoGo dominant foci | | 1 | | | TAL |  |
| Konishi et al (1998) | | 4/1 | 1.5 | E | Go/NoGo, 50% NoGo trials | | | NoGo > Go, NoGo dominant foci | | 19 | | | TAL |  |
| Kuhn et al (2009) | | 5/11 | 3 | B | Go/NoGo, 14% NoGo trials | | | Decide NoGo> Go | | 3 | | | MNI |  |
| Langencker et al (2007) | | 8/14 | 3 | E | Go/NoGo, 20% NoGo trials | | | NoGo > Go, Correct rejection (control) | | 8 | | | TAL |  |
| Laurens et al (2005) | | 5/5 | 1.5 | E | Go/NoGo, 50% NoGo trials | | | NoGo > Go/Rest baseline | | 12 | | | TAL |  |
| Lawrence et al (2009) | | 9/12 | 1.5 | E | Go/NoGo, 12% NoGo trials | | | NoGo > Oddball/Go | | 3 | | | TAL |  |
| Liddle et al (2001) | | 9/7 | 1.5 | E | Go/NoGo, 50% NoGo trials | | | Correct NoGo > Baseline; | | 19 | | | TAL |  |
|  |  |  |  |  |  |  |  | Correct NoGo > Go | | 23 | | |  |  |
| Luetcke et al (2008) | | 8/3 | 2.9 | E | Go/NoGo, 20% NoGo trials | | | Successful NoGo > UnSuccessful Go | | 2 | | | MNI |  |
| Magyire (2003) | | 6 | 1.5 | B | Go/NoGo, 50% NoGo trials | | | Go/NoGo > Go | | 6 | | | TAL |  |
|  |  |  |  |  |  |  |  | Go/NoGo > Control | | 9 | | |  |  |
| Maltby et al (2005) | | 14 | 1.5 | E | Go/NoGo, 17% NoGo trials | | | Correct NoGo > Go | | 5 | | | TAL |  |
| Mazzola-Pomietto et al (2009) | | 16 | 3 | E | Go/NoGo, 50% NoGo trials | | | NoGo > Go (control) | | 7 | | | TAL |  |
| McNab et al (2008) | | 4/7 | 1.5 | E | Go/NoGo, 25% NoGo trials | | | NoGo > Oddball | | 17 | | | MNI |  |
|  |  |  |  |  |  |  |  | NoGo > Go | | 6 | | |  |  |
|  |  |  |  |  |  |  |  | NoGo & Stop > Oddball | | 7 | | |  |  |
|  |  |  |  |  |  |  |  | NoGo & Stop > Go | | 6 | | |  |  |
| Menon et al (2001) | | 8/6 | 1.5 | B | Go/NoGo, 50% NoGo trials | | | NoGo > Go | | 13 | | | TAL |  |
| Mostofsky et al (2003) | | 48 | 1.5 | E | Go/NoGo, 18% NoGo trials | | | NoGo > Fixation (simple) | | 3 | | | TAL |  |
|  |  |  |  |  |  |  |  | NoGo > Fixation (Counting) | | 3 | | |  |  |
| Nakata et al (2008) | | 7/8 | 1.5 | E | Go/NoGo, 50% NoGo trials | | | Movement NoGo > Go | | 10 | | | TAL |  |
|  |  |  |  |  |  |  |  | Cunt NoGo > Go | | 35 | | |  |  |
| Nakata et al (2008) | | 7/8 | 1.5 | E | Go/NoGo, 50% NoGo trials | | | Movement/ Count NoGo > Baseline | | 58 | | | TAL |  |
| Nakata et al (2009) | | 7/8 | 1.5 | E | Go/NoGo, 50% NoGo trials | | | Movement and Count conjunction | | 2 | | | TAL |  |
|  |  |  |  |  |  |  |  | Movement > Baseline | | 13 | | |  |  |
|  |  |  |  |  |  |  |  | Count NoGo > Baseline | | 5 | | |  |  |
| O'Connor et al (2012) | | 9/9 | 3 | B | Go/NoGo, 14% NoGo trials | | | Successful NoGo > Go | | 19 | | | MNI |  |
| Roth et al (2007) | | 6/8 | 1.5 | E | Go/NoGo, 50% NoGo trials | | | NoGo > Go (control) | | 13 | | | TAL |  |
| Rubia et al (2001) | | 15 | 1.5 | B | Go/NoGo, 30% NoGo trials | | | NoGo > Go | | 12 | | | TAL |  |
|  |  |  |  |  |  |  |  | Common regions to Go/NoGo and Stop signal | | 9 | | |  |  |
| Rubia et al (2007) | | 21 | 1.5 | E | Go/NoGo, 20% NoGo trials | | | NoGo > Go (adults) | | 6 | | | TAL |  |
| Rubia et al (2006) | | 23/0 | 1.5 | E | Go/NoGo, 12% NoGo trials | | | NoGo > Go (adults) | | 11 | | | TAL |  |
| Schulz et al (2009) | | 11/13 | 3 | B | Go/NoGo, 25% NoGo trials | | | Correct NoGo > Correct Go | | 12 | | | TAL |  |
| Sebastian et al (2012) | | 11/13 | 3 | E | Go/NoGo, 29% NoGo trials | | | NoGo > Go | | 19 | | | MNI |  |
| Sebastian et al (2013) | | 12/9 | 3 | E | Hybrid Respo nse Inhibition task | | | NoGo > Congruent Go | | 17 | | | MNI |  |
|  |  |  |  |  | Go/NoGo, 29% NoGo trials | | | NoGo > Congruent Go | | 25 | | |  |  |
|  |  |  |  |  | Conjunction analysis | | | Successful inhibition > Go | | 17 | | |  |  |
| Shafritz et al (2015) | | 15/3 | 3 | B | Emotional Go/NoGo, 50% NoGo trials  Nonemotional Go/NoGo, 50% NoGo trials | | | ‘X’ NoGo > Letter Go (control) | | 5 | | | MNI |  |
|  |  |  |  |  |  |  |  | Happy NoGo > Neutral Go (control) | | 6 | | |  |  |
|  |  |  |  |  |  |  |  | Fear NoGo > Neutral Go (control) | | 5 | | |  |  |
|  |  |  |  |  |  |  |  | Happy NoGo > Fear Go (control) | | 8 | | |  |  |
|  |  |  |  |  |  |  |  | Fear NoGo > Happy Go (control) | | 2 | | |  |  |
| Steele et al (2013) | | 49/53 | 3 | E | Go/NoGo, 16% NoGo trials | | | Successful NoGo > Successful Go | | 36 | | | MNI |  |
| Tamm et al (2004) | | 12/0 | 1.5 | E | Go/NoGo, 17% NoGo trials | | | NoGo > Go, controls | | 3 | | | TAL |  |
| Todd et al (2012) | | 8/7 | 1.5 | E | Go/NoGo, 50% NoGo trials | | | NoGo > Go | | 1 | | | TAL |  |
| Townsend et al (2012) | | 17/13 | 3 | E | Go/NoGo, 50% NoGo trials | | | NoGo > Go (control) | | 24 | | | MNI |  |
| van Gaal et al (2010) | | 24 | 3 | E | masked Go/NoGo task, 25% NoGo trials | | | Weakly masked NoGo > Go | | 29 | | | MNI |  |
|  |  |  |  |  |  |  |  | Strongly masked NoGo > Go | | 10 | | |  |  |
| Vanderhasselt et al (2011) | | 34 | 3 | B | Go/NoGo, 20% NoGo trials | | | Correct NoGo > Null event | | 8 | | | MNI |  |
| Vercammen et al (2012) | | 11/12 | 3 | B | Emotional Go/NoGo, 20% NoGo trials | | | Inhibit negative > Inhibit neutral (control) | | 11 | | | MNI |  |
| Vollm et al (2004) | | 8/0 | 1.5 | B | Go/NoGo, 50% NoGo trials | | | NoGo > Go (control) | | 13 | | | TAL |  |
| Wager et al (2005) | | 14 | 3 | B | Go/NoGo, 20% NoGo trials | | | NoGo > Go | | 13 | | | TAL |  |
|  |  |  |  |  |  |  |  | NoGo > Go, unique region | | 12 | | |  |  |
|  |  |  |  |  |  |  |  | Conjunction analysis | | 12 | | |  |  |
| Walther et al (2010) | | 8/9 | 3 | B | Go/NoGo, 20% NoGo trials | | | Conjunction analysis, NoGo > Go | | 31 | | | MNI |  |
| Watanabe et al (2002) | | 9/2 | 1.5 | E | Go/NoGo, 50% NoGo trials | | | NoGo > Go | | 5 | | | TAL |  |
|  |  |  |  |  |  |  |  | NoGo > Go, specific activation areas | | 4 | | |  |  |
| Welander-Vatn et al (2009) | | 11/17 | 1.5 | B | Go/NoGo, 25% NoGo trials | | | Go/NoGo > Fixation (control) | | 12 | | | MNI |  |
| Zheng et al (2008) | | 8/12 | 1.5 | E | Go/NoGo, 25% NoGo trials | | | Successful NoGo > Go | | 8 | | | TAL |  |
|  |  |  |  |  |  |  |  | Common regions | | 2 | | |  |  |
| Nielson et al (2002) | | 34 | 1.5 | E | Go/Nogo, 17.5% nogo trials | | | NoGo > Go | | 42 | | | TAL |  |
| Booth et al (2003) | | 5/7 | 1.5 | B | Go/NOGo, 50% Nogo trials | | | NoGo > Go | | 15 | | | MNI |  |
| Kaufman et al (2003) | | 4/10 | 1.5 | E | Go/NoGo, 6% Nogo trials | | | Successful NoGo > Baseline | | 15 | | | TAL |  |
| Langenecker et al (2003) | | 7/15 | 1.5 | B | Go/nogo, 21% Nogo trials | | | NoGo > Go | | 23 | | | TAL |  |
| Hester et al (2004) | | 7/8 | 1.5 | E | GoNoGo, 12% Nogo trials | | | Successful Nogo >Go | | 29 | | | TAL |  |
| Del ben et al (2005) | | 12 | 1.5 | B | Go/NoGo, 50% NoGo trials | | | NoGo > Go | | 15 | | | MNI |  |
| Talati et al (2005) | | 30 | 1.5 | B | Go/NoGo, 50% NoGo trials | | | NoGo > Go | | 14 | | | TAL |  |
| Schmiz et al (2006) | | 12 |  |  | Go/NoGo, 12% NoGo trials | | | Correct Nogo > Go | | 11 | | | TAL |  |
| Mobbs et al (2007) | | 2/9 | 1.5 | B | Go/NoGo, 50% NoGo trials | | | Nogo >Go | | 4 | | | MNI |  |
| Falconer et al (2008) | | 10/13 | 1.5 | E | Go/Nogo, 25% NoGo trials | | | NoGo > Go | | 6 | | | MNI |  |
| Shane et al (2008) | | 15/6 | 3 | B | Go/NoGo, 20 NoGo trials | | | Successful NoGo > Uncorrected Nogo | | 8 | | | MNI |  |
| Shane et al (2008) | | 15/6 | 3 | B | Observating NoGo 20% NoGo trials | | | Successful NoGo > Uncorrected Nogo | | 4 | | | MNI |  |
| Bernal et al (2009) | | 10/8 | 1.5 | E | Go/NoGo, 50% NoGo trials | | | NoGo >Go | | 38 | | | TAL |  |
| Cojan et al (2009) | | 30 | 1.5 | B | Go/NoGo, 25% NoGo trials | | | NoGo > Go | | 8 | | | MNI |  |
| Page et al (2009) | | 10 | 1.5 | E | Go/NoGo, 12% NoGo trials | | | Nogo >Go | | 3 | | | TAL |  |
| Jamadar et al (2010) | | 24 | 3 | B | Go/NoGo, 33% NoGo trials | | | NoGo>Go | | 43 | | | TAL |  |
| Singth et al (2010) | | 22 | 3 | B | Go/NoGo, 50% NoGo trials | | | NoGo > Go | | 2 | | | MNI |  |
| Rothmayr et al (2011) | | 7/5 | 3 | E | Go/NoGo, 20% NoGo trials | | | Nogo >Go | | 4 | | | MNI |  |
| Schulz et al (2011) | | 8/8 | 3 | B | Go/NoGo, 25% NoGo trials | | | Nogo >Go | | 12 | | | MNI |  |
| Vidal et al (2012) | | 7/7 | 3 | B | Go/NoGo, 34% NoGo trials | | | NoGo > Go | | 6 | | | MNI |  |
| Sebastian et al (2013) | | 49 | 3 | E | Go/NoGo, 29% NoGo trials | | | NoGo > Go | | 26 | | | MNI |  |
| Welander-Vatn et al (2013) | | 24 | 1.5 | B | Go/NoGo, 25% NoGo trials | | | NoGo > Go | | 19 | | | MNI |  |
| Chen et al (2015) | | 25 | 3 | E | Go/Nogo, 17% NoGo trials | | | Nogo >Go | | 7 | | | MNI |  |
| O’Connor et al (2015) | | 12/5 | 3 | E | Go/Nogo, 14% NoGo trials | | | NoGo > Go | | 12 | | | MNI |  |
| Penfold et al (2015) | | 10/10 | 3 | B | Go/Nogo,25% NoGo trials | | | NoGo > Go | | 30 | | | MNI |  |
| Wen et al (2015) | | 18/18 | 3 | E | Go/Nogo, 25% NoGo trials | | | NoGo > Go | | 16 | | | TAL |  |
| Ye et al (2015) | | 8/12 | 3 | E | Go/nogo, 8% NoGo trials | | | NoGo > Go | | 8 | | | MNI |  |
| Ye et al (2015) | | 8/12 | 3 | E | Go/nogo, 8% NoGo trials | | | Successful NoGo > Go | | 10 | | | MNI |  |

| ***Action Cancellation*** | | | | | | | | |
| --- | --- | --- | --- | --- | --- | --- | --- | --- |
| Aron et al (2006) | 9/4 | 3 | B | Stop signal, 25% stop trials | Stop inhibit > Go | | 35 | MNI |
| Aron et al (2007) | 10/5 | 3 | B | Stop signal, 25% stop trials | Critical Stop inhibit > Critical Go | | 38 | MNI |
| Boecker et al (2011) | 15/0 | 1.5 | E | Stop change, 20% stop trials | Stop Inhibit > Go | | 13 | TAL |
| Boehler et al (2011) | 6/9 | 3 | E | Stop signal, 20% stop trials . | All Stop trials > Go trials | | 10 | MNI |
|  |  |  |  |  | Stop-irrelevant stop trials > Stop-irrelevant Go | | 7 |  |
| Boehler et al (2010) | 6/9 | 3 | E | Stop signal, 20% stop trials . | Successful Stop trials > Go-trials | | 30 | MNI |
|  |  |  |  |  | Successful stop > Unsuccessful stop | | 3 |  |
|  |  |  |  |  | Successful stop > Go and UnSuccessful stop>Go | | 23 |  |
|  |  |  |  |  | Successful Stop > control block Stop | | 13 |  |
| Boehler et al (2014) | 1/15 | 3 | E | Stop signal, infrequent stop trials | Stop > Go | | 29 | MNI |
|  |  |  |  |  | Successful stop> UnSuccessful stop | | 4 |  |
| Cai et al (2014) | 11/12 | 3 | B | Stop signal, 30% stop signal | Stop > Go | | 17 | MNI |
|  |  |  |  |  | Successful stop > Go | | 15 |  |
| Cai et al (2009) | 6/6 | 3 | E | Stop signal, 30% stop signal | Stop > Go (color task) | | 8 | MNI |
|  |  |  |  |  | Stop > Go (orientation task) | | 14 |  |
| Cai et al (2011) | 15/11 | 3 | Mixed | Stop signal, 30% stop signal | SST-all stop > SST-Go | | 29 | MNI |
|  |  |  |  |  | Successful stop > Go | | 21 |  |
|  |  |  |  |  | Successful stop > Unsuccessful stop | | 24 |  |
| Chamberlain et al (2009) | 20/0 | 3 | E | Stop signal, 20% stop trials | Successful Stop> Go | | 33 | MNI |
| Chevrier et al (2007) | 8/6 | 1.5 | E | Stop signal, 33% stop trials | Successful Stop > Go | | 3 | MNI |
| Chikaze et al (2009) | 10/12 | 1.5 | E | Stop signal, 33% stop trials | Stop> Uncertain Go trials | | 57 | TAL |
|  |  |  |  |  | Stop>Uncertain-Go trials, unique region | | 16 |  |
| Matthews et al (2005) | 9/7 | 3 | E | Stop signal, 25% stop trials | Hard > easy inhibit trials | | 6 | TAL |
| Ganos et al (2014) | 15 | 3 | B | Stop signal, 25% stop trials | Stop > Go | | 10 | MNI |
| Goghari et al (2009) | 7/5 | 3 | E | Stop signal, Probe related activity | Stop > Go | | 25 | MNI |
| Hu et al (2012) | 46/45 | 3 | E | Stop signal, 25% stop trials | Successful Stop >Successful Go | | 2 | MNI |
| Hughes et al (2014) | 6/6 | 1.5 | B | Stop signal, 33% stop trials | Stop > Go | | 15 | MNI |
|  |  |  |  |  | Stop > Go (Masked) | | 7 |  |
| Hughes et al (2012) | 7/3 | 1.5 | E | Stop signal, 25% stop trials | Stop > Baseline (control) | | 5 | MNI |
| Hughes et al (2013) | 8/7 | 1.5 | B | Stop signal, 30% stop trials | Signal inhibit > Baseline | | 4 | MNI |
|  |  |  |  |  | Signal inhibit > Signal respond | | 2 |  |
| Jahfari et al (2012) | 5/11 | 3 | B | Stop signals, 25% low and 50% high probability stop trials | Low/high probability successful stop > Go | | 8 | MNI |
| Jahfari et al (2012) | 9/11 | 3 | E | Stop signals, 33% stop trials | Successful stop > Go | | 7 | MNI |
| Karoly et al (2014) | 24/29 | 3.0 | E | Stop signal, 26% stop trials | Correct response > Correct Go | | 7 | MNI |
| Lenartowicz (2009) | 8/15 | 3 | B | Stop signal, 24% stop trials | Stop > Go | | 7 | MNI |
| Leung et al (2007) | 6/6 | 3 | E | Stop signal, 30% stop trials | Conjunction analysis, eye and hand, Stop > Go | | 7 | MNI |
| Li et al (2006) | 18/6 | 3 | E | Stop signal, 25% stop trials | Successful stop > Unsuccessful stop | | 9 | TAL |
| Li et al (2008) | 20/20 | 3 | E | Stop signal, 25% stop trials | Successful stop > Go | | 2 | TAL |
|  |  |  |  |  | Short > Long stop processing time | | 5 |  |
| McNab et al (2008) | 4/7 | 1.5 | E | Stop signal, 25% stop trials | Stop > Oddball | | 25 | MNI |
|  |  |  |  |  | Stop > Go | | 16 |  |
|  |  |  |  |  | NoGo & Stop > Oddball | | 7 |  |
|  |  |  |  |  | NoGo & Stop > Go | | 6 |  |
| Ness et al (2013) | 5/8 | 3 | B | Stop change | Stop signal delay 300 > Go | | 9 | MNI |
|  |  |  |  |  | Stop signal delay 300 > Stop signal delay 0 | | 2 |  |
| Padmala et al (2010) | 16/19 | 3 | E | Stop signal, 16% stop trials | Successful > Unsuccessful stop trials | | 14 | TAL |
| Padmala et al (2010) | 16/19 | 3 | E | Stop signal, 16% stop trials | Successful >Unsuccessful stop trials | | 12 | TAL |
| Ramautar et al (2003) | 8/8 | 1.5 | E | Stop signal, 25% low and 50% high probability stop trials | Successful stop > Go | | 7 | TAL |
| Rosell-Negre et al (2014) | 23/5 | 1.5 | E | Stop signal, 30% NoGo trials | Stop> Go | | 9 | MNI |
| Rubia et al (2001) | 15 | 1.5 | B | Stop signal, 30% stop trials | Stop > Go | | 5 | TAL |
|  |  |  |  |  | Activation common to Go/NoGo and Stop signal | | 9 |  |
| Rubia et al (2003) | 20 | 1.5 | E | Stop signal, 20% stop trials | Successful stop > Unsuccessful stop | | 2 | TAL |
| Sagaspe et al (2011) | 14 | 1.5 | E | Stop signal, 33% stop trials | (Stop Inhibit/Respond) > Go | | 25 | MNI |
|  |  |  |  |  | Successful stop > UnSuccessful stop | | 14 |  |
| Schel et al (2014) | 11/13 | 3 | E | Stop signal, 25% stop trials | Successful stop > Go | | 13 | MNI |
| Sebastian et al (2012) | 11/13 | 3 | E | Stop signal, 25% stop trials | Stop > Go | | 28 | MNI |
| Sebastian et al (2013) | 12/9 | 3 | E | Hybrid Respo nse Inhibition task | Stop > Congruent Go | | 24 | MNI |
|  |  |  |  | Stop signal, 25% stop trials | Stop > Congruent Go | | 22 | MNI |
|  |  |  |  | Conjunction analysis | Successful inhibition > Go | | 17 | MNI |
| Sharp et al (2010) | 17/9 | 3 | E | Stop signal, 20% stop trials | Correct stop > Go | | 10 | MNI |
|  |  |  |  |  | Correct stop > Continue | | 6 |  |
| Strakowski et al (2008) | 9/7 | 4.0 | E | Stop signal, 25% stop trials | Correct stop > Correct Go (control) | | 18 | TAL |
| Tabu et al (2011) | 8/5 | 3 | E | Stop signal, 25% stop trials | Successful stop> Go | | 6 | MNI |
|  |  |  |  | Double-press signal, 25% stop trials | Successful stop > Go | | 2 | MNI |
|  |  |  |  |  | Conjunction analysis | | 2 |  |
| Tabu et al (2012) | 11/2 | 3 | E | Stop signal, 20% trials | Hand Stop Successful > Go | | 5 | MNI |
| Vink et al (2005) | 10/10 | 1.5 | B | Stop signal, 20% stop trials | Stop > Go | | 4 | MNI |
|  |  |  |  |  | Stop > Go, parametric analysis | | 4 |  |
|  |  |  |  |  | Correct Stop > Incorrect stop | | 2 |  |
| White et al (2014) | 66/57 | 3 | B | Stop signal, 25% stop trials | Stop > Go, RT negative correlation | | 5 | MNI |
|  |  |  |  |  | Stop > Go, Drift positive correlation; | | 6 |  |
|  |  |  |  |  | Stop > Go, SSRT negative correlation | | 5 |  |
|  |  |  |  |  | Stop > Go, Nondecision negative correlation | | 1 |  |
| Wilbertz et al (2014) | 49 | 3 | E | Stop signal, 33% stop trials | Stop > Go | | 45 | MNI |
| Xu et al (2015) | 9/9 | 3 | E | Stop signal, 25% stop trials | Stop > Go | | 16 | MNI |
| Zandbelt et al (2010) | 6/18 | 3 | B | Stop signal, >0% stop trials | StopSuccessful > StopFailure | | 140 | MNI |
| Zheng et al (2008) | 8/12 | 1.5 | E | Stop signal, 25% stop trials | Successful Stop > Go | | 10 | TAL |
|  |  |  |  |  | Common regions | | 2 |  |
| Xue et al (2008) | 6/9 | 3 | E | Stop signal, 25% stop trials | Stop inhibit > Go, Manual | | 13 | MNI |
| Ramautar et al (2005) | 8/8 | 1.5 | B | Stop signal | Successful Stop > Go | | 7 | MNI |
| Li et al (2008) | 20/20 | 3 | E | Stop signal | Stop >Go | | 2 | MNI |
| Li et al (2008) | 20/20 | 3 | E | Stop signal | Successful Stop > Unsuccessful stop | | 17 | MNI |
| Li et al (2008) | 30 | 3 | E | Stop signal | short delay stop > Long delay stop | | 13 | MNI |
| Marco-Pallares | 3/7 | 1.5 | E | Stop signal | Correct stop > Go | | 9 | MNI |
| Chao et al (2009) | 30/35 | 3 | E | Stop signal | short delay stop > Long delay stop | | 3 | MNI |
| Hendrick et al (2010) | 30/30 | 3 | E | Stop | Stop > Go | | 17 | MNI |
| Li et al (2010) | 36 | 3 | E | Stop signal | Successful Stop > unsuccessful stop | | 15 | MNI |
| Passarotti et al (2010) | 15 | 3 | B | Stop signal | Stop > Go | | 5 | TAL |
| Dodds et al (2011) | 13/7 | 3 | B | Stop signal | Stop > Go | | 4 | MNI |
| Bobb et al (2012) | 4/9 | 3 | E | Stop signal | Stop >Go | | 6 | MNI |
| Swann et al (2012) | 8/8 | 3 | E | Stop signal | Successful stop >Go | | 18 | MNI |
| Zhang et al (2012) | 10/15 | 3 | E | Stop signal | Stop successful >Unsuccessful stop | | 3 | MNI |
| Majid et al (2013) | 8/10 | 3 | E | Stop signal | Outright Stop > Go | | 20 | MNI |
| Majid et al (2013) | 8/10 | 3 | E | Stop signal | Prepare Stop > Go | | 14 | MNI |
| Sebastian et al (2013) | 49 | 3 | E | Stop signal | Stop > Go | | 19 | MNI |
| Zandbelt et al (2013) | 9/13 | 3 | B | Stop signal | Stop > Go | | 35 | MNI |
| Congdon et al (2014) | 30/32 | 3 | B | Stop signal | Stop > Go | | 4 | MNI |
| Ganos et al (2014) | 15 | 3 | B | Stop signal | Stop > Go | | 10 | MNI |
| Lavalee et al (2014) | 17/20 | 1.5 | B | Stop signal | Stop > Go | | 9 | MNI |
| Mohammadi et al (2015) | 10/7 | 3 | B | Stop signal | Successful stop>unsuccessful nogo | | 13 | MNI |
| Mohammadi et al (2015) | 10/7 | 3 | B | Stop signal | Successful stop >Go | | 11 | MNI |
| Coxon et al (2015) | 9/11 | 3 | B | Stop signal | Stop > Go | | 29 | MNI |
| Sebastian et al (2015) | 11/17 | 3 | E | Stop signal | Stop > Go | | 13 | MNI |
| Sebastian et al (2015) | 11/17 | 3 | E | Stop signal | Stop > Attention capture | | 11 | MNI |
| Cummins et al (2012) | 100 | 1.5 | B | Stop signal | Successful inhibition >Go | | 5 | MNI |
| Jahfari et al (2011) | 9/11 | 3 | B | Stop signal | Successful inhibition >Go | | 7 | MNI |
| Brown et al (2005) | 16 | 1.5 | B | Stop siganl | Stop> Go | 8 | | TAL |
| Rae et al (2015)  Coxon et al (2009) | 9/11  8/5 | 3  1.5 | B  E | Stop signal  Stop signal | Successful inhibition >Go  Stop > Go | | 59  30 | MNI  MNI |

Note: E: event related experiment design; B: block experiment design; M/F: Male/Female; MNI: Montreal Neurological Institute's space; TAL: Talairach space. WCST: Wisconsin Card Sorting Test.

Table S3 Brain areas activated by all studies (*p* < 0.05, family-wise error (FWE) corrected across the whole brain).

| Region | R/L | Center (x, y, z) | | | Maximum P | No. Voxs |
| --- | --- | --- | --- | --- | --- | --- |
| Insula | R | 36 | 20 | 12 | 0.29 | 5633 |
| Thalamus | R | 6 | -12 | 0 | 0.13 |  |
| Pallidum | R | 18 | 8 | 2 | 0.15 |  |
| Inferior frontal gyrus, triangular part | R | 46 | 38 | 6 | 0.15 |  |
| Middle frontal gyrus | R | 40 | 40 | 26 | 0.18 |  |
| Inferior frontal gyrus, opercular part | R | 46 | 12 | 30 | 0.21 |  |
| Precentral gyrus | R | 40 | 4 | 46 | 0.15 |  |
| Angular gyrus | R | 48 | -46 | 32 | 0.21 | 2995 |
| Superior temporal gyrus | R | 54 | -24 | -4 | 0.11 |  |
| Middle temporal gyrus | R | 58 | -34 | 2 | 0.13 |  |
| Supramarginal gyrus | R | 54 | -44 | 36 | 0.21 |  |
| Angular gyrus | R | 34 | -56 | 48 | 0.17 |  |
| Supplementary motor area | R | 4 | 16 | 46 | 0.26 | 2297 |
| Middle cingulate gyrus | R | 4 | 28 | 32 | 0.20 |  |
| Insula | L | -36 | 18 | -2 | 0.26 | 1172 |
| Putamen | L | -16 | 8 | 4 | 0.15 | 706 |
| Inferior parietal gyrus | L | -30 | -56 | 48 | 0.13 | 561 |
| Inferior temporal gyrus | L | -42 | -64 | -10 | 0.14 | 409 |
| Supramarginal gyrus | L | -58 | -48 | 24 | 0.12 | 353 |
| Precentral gyrus | L | -42 | 2 | 38 | 0.10 | 212 |
| Middle frontal gyrus | L | -30 | -4 | 52 | 0.09 |  |
| Inferior frontal gyrus, triangular part | L | -42 | 24 | 30 | 0.11 | 114 |
| Middle frontal gyrus | L | -34 | 48 | 22 | 0.11 | 54 |
| Middle cingulate gyrus | R | 2 | -22 | 30 | 0.10 | 53 |
| Middle occipital gyrus | L | -30 | -90 | -4 | 0.09 | 15 |

Note: R/L, right/left hemisphere; Maximum P is the maximum proportion of studies exhibiting the effect at the peak density weighted by sample size. Coordinates are Montreal Neurological Institute standard stereotaxic spaces. The voxel size is 2x2x2 mm^3^.

Table S4 Brain areas activated by 68 randomly selected contrasts from action withholding and action cancellation papers, respectively (p < 0.05, FWE corrected across the whole brain).

| Region | R/L | Center (x, y, z) | | | Maximum P | No. Voxs |
| --- | --- | --- | --- | --- | --- | --- |
| ***Action withholding*** |  |  |  |  |  |  |
| Inferior frontal gyrus, triangular part | R | 46 | 28 | 24 | 0.26 | 586 |
| Inferior frontal gyrus, opercular part | R | 50 | 18 | 28 | 0.22767 |  |
| Insula | R | 36 | 24 | -2 | 0.25 | 345 |
| Supplementary motor area | R | 6 | 10 | 54 | 0.23 | 291 |
| Inferior parietal gyrus | R | 50 | -44 | 40 | 0.21 | 187 |
| Insula | L | -34 | 18 | -2 | 0.21 | 103 |
| Caudate | R | 14 | 6 | 8 | 0.2 | 99 |
| Superior parietal gyrus | R | 30 | -64 | 52 | 0.18 | 55 |
| Middle temporal gyrus | R | 58 | -48 | 8 | 0.19 | 33 |
| Middle temporal gyrus | R | 58 | -28 | -4 | 0.16 | 17 |
| Superior temporal gyrus | R | 56 | -46 | 20 | 0.17 | 17 |
| ***Action cancellation*** |  |  |  |  |  |  |
| Insula | R | 40 | 18 | -2 | 0.39 | 1418 |
| Putamen | R | 22 | 12 | 0 | 0.25 |  |
| Inferior frontal gyrus, opercular part | R | 52 | 18 | 4 | 0.28 |  |
| Insula | L | -34 | 16 | -2 | 0.42 | 1028 |
| Insula | L | -38 | 18 | -4 | 0.42 |  |
| Putamen | L | -16 | 10 | 2 | 0.21 |  |
| Supplementary motor area | R | 4 | 18 | 50 | 0.35 | 894 |
| Middle cingulate gyrus | R | 4 | 28 | 34 | 0.22 |  |
| Inferior parietal gyrus | R | 50 | -44 | 38 | 0.24 | 747 |
| Supramarginal gyrus | R | 60 | -44 | 24 | 0.21 |  |
| Inferior parietal gyrus | R | 34 | -52 | 48 | 0.24 |  |
| Inferior frontal gyrus, opercular part | R | 46 | 10 | 32 | 0.23 | 344 |
| Middle frontal gyrus | R | 32 | 46 | 28 | 0.24 | 237 |
| Thalamus | R | 8 | -14 | 0 | 0.19 | 26 |

Note: R/L, right/left hemisphere; Maximum P is the maximum proportion of studies exhibiting the effect at the peak density weighted by sample size. Coordinates are Montreal Neurological Institute standard stereotaxic spaces. The voxel size is 2x2x2 mm^3^.

Table S5 Brain areas activated by all randomly select studies (*p* < 0.05, FWE corrected across the whole brain).

| Region | R/L | Center (x, y, z) | | | Maximum P | No. Voxs |
| --- | --- | --- | --- | --- | --- | --- |
| Inferior frontal gyrus, opercular part | R | 38 | 18 | 12 | 0.29 | 3684 |
| Insula | R | 40 | 20 | -4 | 0.29 |  |
| Thalamus | R | 6 | -12 | 4 | 0.14 |  |
| Pallidum | R | 18 | 8 | 4 | 0.15 |  |
| Inferior frontal gyrus, triangular part | R | 50 | 20 | 20 | 0.17 |  |
| Middle frontal gyrus | R | 40 | 40 | 24 | 0.16 |  |
| Precentral gyrus | R | 38 | 2 | 48 | 0.14 |  |
| Supplementary motor area | R | 4 | 16 | 48 | 0.30 | 1789 |
| Inferior parietal gyrus | R | 44 | -48 | 40 | 0.18 | 1762 |
| Superior temporal gyrus | R | 58 | -46 | 14 | 0.18 |  |
| Supramarginal gyrus | R | 52 | -42 | 36 | 0.18 |  |
| Superior parietal gyrus | R | 32 | -56 | 48 | 0.16 |  |
| Insula | L | -30 | 16 | 0 | 0.24 | 1252 |
| Superior parietal gyrus | L | -26 | -58 | 50 | 0.15 | 464 |
| Inferior parietal gyrus | L | -34 | -50 | 46 | 0.13 |  |
| Superior parietal gyrus | L | -26 | -60 | 50 | 0.15 |  |
| Middle frontal gyrus | L | -28 | -2 | 54 | 0.12 | 178 |
| Inferior frontal gyrus, triangular part | L | -42 | 24 | 28 | 0.12 | 174 |
| Inferior occipital gyrus | L | -40 | -64 | -10 | 0.12 | 160 |
| Middle temporal gyrus | R | 58 | -30 | -2 | 0.1 | 37 |
| Inferior parietal gyrus | L | -46 | -38 | 46 | 0.11 | 29 |
| Inferior occipital gyrus | L | -44 | -78 | -4 | 0.1 | 10 |

Note: R/L, right/left hemisphere; Maximum P is the maximum proportion of studies exhibiting the effect at the peak density weighted by sample size. Coordinates are Montreal Neurological Institute standard stereotaxic spaces. The voxel size is 2x2x2 mm^3^.

Table S6 Brain areas activated by low (<50%) versus high (50%) frequencies of NoGo stimulus from the MKDA analysis (p < 0.05, FWE corrected across the whole brain).

| Region | R/L | Center (x, y, z) | | | Maximum P | No. Voxs |
| --- | --- | --- | --- | --- | --- | --- |
| ***High frequency NoGo (=50%)*** |  |  |  |  |  |  |
| Supplementary motor area | R | 6 | 18 | 46 | 0.38 | 281 |
| Insula | L | -40 | 16 | 6 | 0.30 | 161 |
| Insula | R | 38 | 22 | 4 | 0.30 | 136 |
| Angular gyrus | R | 58 | -50 | 30 | 0.26 | 41 |
| Middle frontal gyrus | R | 40 | 36 | 24 | 0.26 | 29 |
| Inferior frontal gyrus, opercular part | R | 48 | 12 | 34 | 0.27 | 15 |
| ***Low frequency NoGo (< 50%)*** |  |  |  |  |  |  |
| Inferior frontal gyrus, triangular part | R | 42 | 30 | 26 | 0.23 | 1302 |
| Middle frontal gyrus | R | 38 | 42 | 26 | 0.23 |  |
| Precentral gyrus | R | 46 | 8 | 38 | 0.18 |  |
| Supramarginal gyrus | R | 52 | -44 | 32 | 0.21 | 1006 |
| Superior temporal gyrus | R | 56 | -48 | 14 | 0.18 |  |
| Insula | R | 38 | 20 | -4 | 0.26 | 756 |
| Supplementary motor area | R | 4 | 12 | 50 | 0.19 | 635 |
| Middle cingulate gyrus | R | 2 | 20 | 40 | 0.17 |  |
| Middle temporal gyrus | R | 56 | -30 | -2 | 0.2 | 255 |
| Superior parietal gyrus | R | 30 | -62 | 50 | 0.18 | 250 |
| Superior parietal gyrus | L | -26 | -60 | 50 | 0.17 | 159 |
| Fusiform gyrus | L | -40 | -62 | -12 | 0.16 | 143 |
| Pallidum | R | 20 | 8 | 4 | 0.17 | 137 |
| Middle frontal gyrus | L | -34 | 48 | 22 | 0.14 | 20 |

Note: R/L, right/left hemisphere; Maximum P is the maximum proportion of studies exhibiting the effect at the peak density weighted by sample size. Coordinates are Montreal Neurological Institute standard stereotaxic spaces. The voxel size is 2x2x2 mm^3^.

Table S7 Brain areas activated by NoGo vs Go trials only from the MKDA analysis (p < 0.05 FWE corrected across the whole brain).

| Region | R/L | Center (x, y, z) | | | Maximum P | No. Voxs |
| --- | --- | --- | --- | --- | --- | --- |
| Inferior frontal gyrus, triangular part | R | 40 | 24 | 14 | 0.26 | 2526 |
| Insula | R | 34 | 18 | -10 | 0.24103 |  |
| Pallidum | R | 20 | 8 | 2 | 0.15502 |  |
| Inferior frontal gyrus, triangular part | R | 46 | 30 | 20 | 0.18879 |  |
| Middle frontal gyrus | R | 38 | 42 | 26 | 0.23212 |  |
| Inferior frontal gyrus, opercular part | R | 48 | 14 | 28 | 0.2118 |  |
| Precentral gyrus | R | 44 | 6 | 42 | 0.17416 |  |
| Supramarginal gyrus | R | 48 | -46 | 34 | 0.21 | 1593 |
| Middle temporal gyrus | R | 58 | -32 | -2 | 0.17172 |  |
| Superior temporal gyrus | R | 56 | -46 | 14 | 0.15985 |  |
| Angular gyrus | R | 32 | -58 | 48 | 0.17783 |  |
| Supplementary motor area | R | 4 | 14 | 48 | 0.19 | 742 |
| Middle cingulate gyrus | R | 4 | 16 | 44 | 0.19176 |  |
| Precentral gyrus | L | -44 | 0 | 46 | 0.16 | 40 |
| Superior parietal gyrus | L | -24 | -60 | 52 | 0.14 | 39 |
| Middle frontal gyrus | L | -32 | 50 | 24 | 0.13 | 28 |
| Inferior parietal lobule | L | -42 | -48 | 44 | 0.13 | 18 |
| Middle frontal gyrus | R | 30 | 4 | 56 | 0.13 | 14 |
| Fusiform gyrus | L | -38 | -60 | -12 | 0.13 | 11 |

Note: R/L, right/left hemisphere; Maximum P is the maximum proportion of studies exhibiting the effect at the peak density weighted by sample size. Coordinates are Montreal Neurological Institute standard stereotaxic spaces. The voxel size is 2x2x2 mm^3^.

Table S8 Brain areas activated by Stop vs Go trials only from the MKDA analysis (p < 0.05, FWE corrected across the whole brain).

| Region | R/L | Center (x, y, z) | | | Maximum P | No. Voxs |
| --- | --- | --- | --- | --- | --- | --- |
| Supplementary motor area | R | 6 | 20 | 48 | 0.27 | 1078 |
| Middle cingulate gyrus | R | 6 | 28 | 36 | 0.23 |  |
| Insula | R | 38 | 20 | -2 | 0.35 | 1071 |
| Insula | L | -36 | 18 | -4 | 0.39 | 824 |
| Supramarginal gyrus | R | 52 | -44 | 38 | 0.29 | 725 |
| Inferior parietal gyrus | R | 36 | -52 | 46 | 0.19 |  |
| Inferior frontal gyrus, opercular part | R | 46 | 10 | 32 | 0.21 | 314 |
| Superior temporal gyrus | L | -58 | -48 | 18 | 0.17 | 208 |
| Middle temporal gyrus | L | -58 | -50 | 10 | 0.17 |  |
| Supramarginal gyrus | L | -60 | -48 | 26 | 0.16 |  |
| Middle frontal gyrus | R | 32 | 46 | 26 | 0.16 | 72 |
| Middle frontal gyrus | R | 46 | 42 | 2 | 0.15 | 64 |
| Fusiform gyrus | L | -40 | -62 | -12 | 0.15 | 61 |
| Caudate | L | -10 | 8 | 4 | 0.17 | 54 |
| Middle temporal gyrus | R | 58 | -42 | 8 | 0.14 | 24 |

Note: R/L, right/left hemisphere; Maximum P is the maximum proportion of studies exhibiting the effect at the peak density weighted by sample size. Coordinates are Montreal Neurological Institute standard stereotaxic spaces. The voxel size is 2x2x2 mm^3^.

Table S9 The distribution of brain activation patterns overlapped on the functional network template.

|  | | Relative distribution (Absolute distribution) | | | | | | | | | | |
| --- | --- | --- | --- | --- | --- | --- | --- | --- | --- | --- | --- | --- |
| Main analyses | | | | | | | | | | | | |
|  | | All | | IR | | | | AW | | | | AC |
| VN | | 2% (1%) | | 0% (0%) | | | | 1% (0%) | | | | 0% (0%) |
| SMN | | 2% (1%) | | 1% (0%) | | | | 1% (0%) | | | | 1% (0%) |
| DAN | | 19% (15%) | | 28% (3%) | | | | 15% (6%) | | | | 8% (3%) |
| VAN | | 27% (25%) | | 45% (5%) | | | | 29% (13%) | | | | 38% (14%) |
| AFN | | 0% (0%) | | 0% (0%) | | | | 0% (0%) | | | | 0% (0%) |
| FPN | | 37% (23%) | | 21% (2%) | | | | 39% (12%) | | | | 35% (9%) |
| DMN | | 13% (5%) | | 4% (0%) | | | | 15% (3%) | | | | 17% (3%) |
| Random select (68 contrast for each classification) | | | | | | | | | | | | |
|  | | All | | IR | | | | AW | | | AC | |
| VN | | 1% (0%) | | -- | | | | 0% (0%) | | | 0% (0%) | |
| SMN | | 1% (1%) | | -- | | | | 1% (0%) | | | 1% (0%) | |
| DAN | | 21% (11%) | | -- | | | | 6% (1%) | | | 10% (3%) | |
| VAN | | 31% (17%) | | -- | | | | 29% (3%) | | | 41% (12%) | |
| AFN | | 1% (0%) | | -- | | | | 0% (0%) | | | 1% (0%) | |
| FPN | | 35% (14%) | | -- | | | | 53% (4%) | | | 32% (6%) | |
| DMN | | 11% (3%) | | -- | | | | 11% (1%) | | | 15% (2%) | |
| AW | | | | | | | | | | | | |
| AW (NoGo - Go only) | | | | | | AW (Low frequency NoGo) | | | | | AW (high frequency NoGo) | |
| VN | | 0% (0%) | | | | 1% (0%) | | | | | 0% (0%) | |
| SMN | | 1% (0%) | | | | 2% (1%) | | | | | 0% (0%) | |
| DAN | | 14% (4%) | | | | 15% (5%) | | | | | 2% (0%) | |
| VAN | | 29% (10%) | | | | 25% (8%) | | | | | 74% (4%) | |
| AFN | | 0% (0%) | | | | 0% (0%) | | | | | 0% (0%) | |
| FPN | | 41% (10%) | | | | 39% (9%) | | | | | 18% (1%) | |
| DMN | | 14% (2%) | | | | 17% (3%) | | | | | 7% (0%) | |
| AC | | | | | | | | | IR | | | |
| AC (Stop –Go only) | | | | | | | | | Exclude SRC | | | Exclude WSCT |
|  | VN | | 1% (0%) | |  | | | | | 0% (0%) | | 0% (0%) |
|  | SMN | | 1% (0%) | |  | | | | | 2% (0%) | | 2% (0%) |
|  | DAN | | 7% (2%) | |  | | | | | 27% (3%) | | 22% (2%) |
|  | VAN | | 39% (11%) | |  | | | | | 45% (5%) | | 51% (6%) |
|  | AFN | | 0% (0%) | |  | | | | | 0% (0%) | | 1% (0%) |
|  | FPN | | 37% (8%) | |  | | | | | 22% (2%) | | 20% (2%) |
|  | DMN | | 16% (2%) | |  | | | | | 5% (0%) | | 4% (0%) |
| IR | | | | | | | | | | | | |
| Exclude Flanker | | | | Exclude Simon | | | Exclude Stroop | | | | Exclude Antiscade | |
| VN | | 0% (0%) | | 0% (0%) | | | 0% (0%) | | | | 0% (0%) | |
| SMN | | 1% (0%) | | 0% (0%) | | | 2% (0%) | | | | 2% (0%) | |
| DAN | | 30% (3%) | | 31% (3%) | | | 35% (2%) | | | | 15% (1%) | |
| VAN | | 44% (4%) | | 38% (4%) | | | 52% (4%) | | | | 48% (4%) | |
| AFN | | 0% (0%) | | 0% (0%) | | | 0% (0%) | | | | 1% (0%) | |
| FPN | | 23% (2%) | | 26% (2%) | | | 10% (1%) | | | | 30% (2%) | |
| DMN | | 3% (0%) | | 5% (0%) | | | 1% (0%) | | | | 5% (0%) | |

Note:VN, visual network; SMN, Sensormotor network; DAN. Dorsal attention network; VAN, ventral attention network; AFN, affective network; FPN, fronto-parietal network; DMN, default mode network. IR: interference resolution; AW, action withholding, and AC, action cancellation; Stimulus response compatibility (SRC); Wisconsin Card Sorting Test (WSCT) tasks.


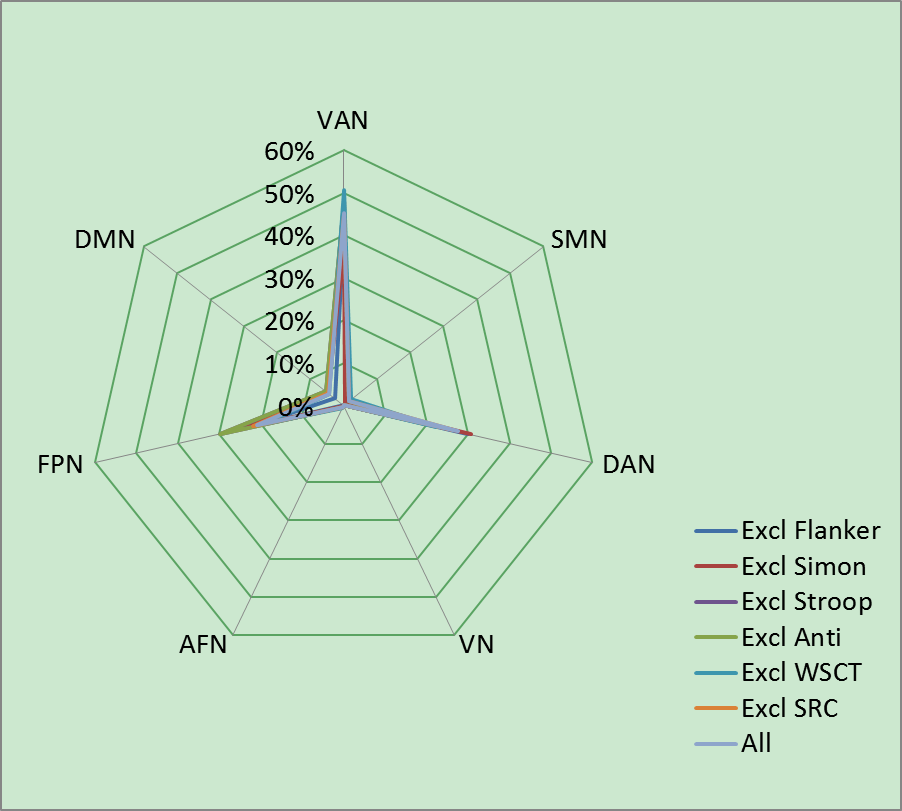


Fig. S1. Using leave out cross validation procedure to test the paradigm effects on detecting the network distribution of interference control. We excluded contrasts of Antiscade task, Flanker task, Simon task, Stimulus response compatibility (SRC), Stroop task, and Wisconsin Card Sorting Test (WSCT) tasks, respectively for each leave out procedure. VAN: ventral attention network; FPN, fronto-parietal network; DMN, default mode network; DAN, dorsal attention network; SMN, sensorimotor network; VN, visual network; AFN, affective network.

**References (Included articles in the meta-analyses)**

***Interference resolution***

Blasi, G., T. E. Goldberg, T. Weickert, S. Das, P. Kohn, B. Zoltick, A. Bertolino, J. H. Callicott, D. R. Weinberger and V. S. Mattay (2006). "Brain regions underlying response inhibition and interference monitoring and suppression." European Journal of Neuroscience **23**(6): 1658-1664.

Brass, M., J. Derrfuss and D. Y. von Cramon (2005). "The inhibition of imitative and overlearned responses: a functional double dissociation." Neuropsychologia **43**(1): 89-98.

Brass, M., S. Zysset and D. Y. von Cramon (2001). "The inhibition of imitative response tendencies." Neuroimage **14**(6): 1416-1423.

Brown, M. R., H. C. Goltz, T. Vilis, K. A. Ford and S. Everling (2006). "Inhibition and generation of saccades: rapid event-related fMRI of prosaccades, antisaccades, and nogo trials." Neuroimage **33**(2): 644-659.

Bunge, S. A., N. M. Dudukovic, M. E. Thomason, C. J. Vaidya and J. D. Gabrieli (2002). "Immature frontal lobe contributions to cognitive control in children: evidence from fMRI." Neuron **33**(2): 301-311.

Chikazoe, J., S. Konishi, T. Asari, K. Jimura and Y. Miyashita (2007). "Activation of right inferior frontal gyrus during response inhibition across response modalities." Journal of Cognitive Neuroscience **19**(1): 69-80.

Ford, K. A., H. C. Goltz, M. R. Brown and S. Everling (2005). "Neural processes associated with antisaccade task performance investigated with event-related FMRI." Journal of Neurophysiology **94**(1): 429-440.

Forstmann, B. U., S. Jahfari, H. S. Scholte, U. Wolfensteller, W. P. van den Wildenberg and K. R. Ridderinkhof (2008). "Function and structure of the right inferior frontal cortex predict individual differences in response inhibition: a model-based approach." The Journal of Neuroscience **28**(39): 9790-9796.

Forstmann, B. U., W. P. van den Wildenberg and K. R. Ridderinkhof (2008). "Neural mechanisms, temporal dynamics, and individual differences in interference control." Journal of Cognitive Neuroscience **20**(10): 1854-1865.

Fukumoto‐Motoshita, M., M. Matsuura, T. Ohkubo, H. Ohkubo, N. Kanaka, E. Matsushima, M. Taira, T. Kojima and T. Matsuda (2009). "Hyperfrontality in patients with schizophrenia during saccade and antisaccade tasks: a study with fMRI." Psychiatry and Clinical Neurosciences **63**(2): 209-217.

Garavan, H., T. Ross and E. Stein (1999). "Right hemispheric dominance of inhibitory control: an event-related functional MRI study." Proceedings of the National Academy of Sciences **96**(14): 8301-8306.

Hazeltine, E., R. Poldrack and J. D. Gabrieli (2000). "Neural activation during response competition." Cognitive Neuroscience, Journal of **12**(Supplement 2): 118-129.

Kim, C., J. K. Kroger and J. Kim (2011). "A functional dissociation of conflict processing within anterior cingulate cortex." Human Brain Mapping **32**(2): 304-312.

Konishi, S., K. Jimura, T. Asari and Y. Miyashita (2003). "Transient activation of superior prefrontal cortex during inhibition of cognitive set." The Journal of Neuroscience **23**(21): 7776-7782.

Konishi, S., T. Watanabe, K. Jimura, J. Chikazoe, S. Hirose, H. M. Kimura and Y. Miyashita (2011). "Role for presupplementary motor area in inhibition of cognitive set interference." Journal of Cognitive Neuroscience **23**(3): 737-745.

Liu, X., M. T. Banich, B. L. Jacobson and J. L. Tanabe (2004). "Common and distinct neural substrates of attentional control in an integrated Simon and spatial Stroop task as assessed by event-related fMRI." Neuroimage **22**(3): 1097-1106.

Matsuda, T., M. Matsuura, T. Ohkubo, H. Ohkubo, E. Matsushima, K. Inoue, M. Taira and T. Kojima (2004). "Functional MRI mapping of brain activation during visually guided saccades and antisaccades: cortical and subcortical networks." Psychiatry Research: Neuroimaging **131**(2): 147-155.

Mayer, A. R., T. M. Teshiba, A. R. Franco, J. Ling, M. S. Shane, J. M. Stephen and R. E. Jung (2012). "Modeling conflict and error in the medial frontal cortex." Human Brain Mapping **33**(12): 2843-2855.

McDowell, J. E., G. G. Brown, M. Paulus, A. Martinez, S. E. Stewart, D. J. Dubowitz and D. L. Braff (2002). "Neural correlates of refixation saccades and antisaccades in normal and schizophrenia subjects." Biological Psychiatry **51**(3): 216-223.

McNab, F., G. Leroux, F. Strand, L. Thorell, S. Bergman and T. Klingberg (2008). "Common and unique components of inhibition and working memory: an fMRI, within-subjects investigation." Neuropsychologia **46**(11): 2668-2682.

Mitchell, R. L. (2005). "The BOLD response during Stroop task-like inhibition paradigms: effects of task difficulty and task-relevant modality." Brain and Cognition **59**(1): 23-37.

Potenza, M. N., H.-C. Leung, H. P. Blumberg, B. S. Peterson, R. K. Fulbright, C. M. Lacadie, P. Skudlarski and J. C. Gore (2014). "An FMRI Stroop task study of ventromedial prefrontal cortical function in pathological gamblers." American Journal of Psychiatry.

Reuter, B., C. Kaufmann, J. Bender, T. Pinkpank and N. Kathmann (2010). "Distinct neural correlates for volitional generation and inhibition of saccades." Journal of Cognitive Neuroscience **22**(4): 728-738.

Rubia, K., A. B. Smith, J. Woolley, C. Nosarti, I. Heyman, E. Taylor and M. Brammer (2006). "Progressive increase of frontostriatal brain activation from childhood to adulthood during event‐related tasks of cognitive control." Human Brain Mapping **27**(12): 973-993.

Schwerdtfeger, R. M. H., N. Alahyane, D. C. Brien, B. C. Coe, P. W. Stroman and D. P. Munoz (2013). "Preparatory neural networks are impaired in adults with attention-deficit/hyperactivity disorder during the antisaccade task." NeuroImage: Clinical **2**: 63-78.

Sebastian, A., B. Gerdes, B. Feige, S. Klöppel, T. Lange, A. Philipsen, L. T. van Elst, K. Lieb and O. Tüscher (2012). "Neural correlates of interference inhibition, action withholding and action cancelation in adult ADHD." Psychiatry Research: Neuroimaging **202**(2): 132-141.

Sebastian, A., M. Pohl, S. Klöppel, B. Feige, T. Lange, C. Stahl, A. Voss, K. Klauer, K. Lieb and O. Tüscher (2013). "Disentangling common and specific neural subprocesses of response inhibition." Neuroimage **64**: 601-615.

Tu, P., T. Yang, W. Kuo, J. Hsieh and T. Su (2006). "Neural correlates of antisaccade deficits in schizophrenia, an fMRI study." Journal of Psychiatric Research **40**(7): 606-612.

Wager, T. D., C.-Y. C. Sylvester, S. C. Lacey, D. E. Nee, M. Franklin and J. Jonides (2005). "Common and unique components of response inhibition revealed by fMRI." Neuroimage **27**(2): 323-340.

Wagner, G., E. Sinsel, T. Sobanski, S. Köhler, V. Marinou, H.-J. Mentzel, H. Sauer and R. G. Schlösser (2006). "Cortical inefficiency in patients with unipolar depression: an event-related FMRI study with the Stroop task." Biological Psychiatry **59**(10): 958-965.

Wingenfeld, K., N. Rullkoetter, C. Mensebach, T. Beblo, M. Mertens, S. Kreisel, M. Toepper, M. Driessen and F. G. Woermann (2009). "Neural correlates of the individual emotional Stroop in borderline personality disorder." Psychoneuroendocrinology **34**(4): 571-586.

Bernal, B. and N. Altman (2009). "Neural networks of motor and cognitive inhibition are dissociated between brain hemispheres: an fMRI study." International Journal of Neuroscience **119**(10): 1848-1880.

Berron, D., S. Frühholz and M. Herrmann (2015). "Neural Control of Enhanced Filtering Demands in a Combined Flanker and Garner Conflict Task." PloS one **10**(3): e0120582.

Erickson, K. I., M. P. Milham, S. J. Colcombe, A. F. Kramer, M. T. Banich, A. Webb and N. J. Cohen (2004). "Behavioral conflict, anterior cingulate cortex, and experiment duration: Implications of diverging data." Human Brain Mapping **21**(2): 98-107.

Grandjean, J., K. D’Ostilio, C. Phillips, E. Balteau, C. Degueldre, A. Luxen, P. Maquet, E. Salmon and F. Collette (2012). "Modulation of brain activity during a Stroop inhibitory task by the kind of cognitive control required." PloS one **7**(7): e41513.

Iannaccone, R., T. U. Hauser, P. Staempfli, S. Walitza, D. Brandeis and S. Brem (2015). "Conflict monitoring and error processing: new insights from simultaneous EEG–fMRI." Neuroimage **105**: 395-407.

King, J. A., F. M. Korb and T. Egner (2012). "Priming of control: implicit contextual cuing of top-down attentional set." The Journal of Neuroscience **32**(24): 8192-8200.

Korsch, M., S. Frühholz and M. Herrmann (2014). "Ageing differentially affects neural processing of different conflict types—an fMRI study." Frontiers in aging neuroscience **6**: 57.

Mead, L. A., A. R. Mayer, J. A. Bobholz, S. J. Woodley, J. M. Cunningham, T. A. Hammeke and S. M. Rao (2002). "Neural basis of the Stroop interference task: response competition or selective attention?" J Int Neuropsychol Soc **8**(6): 735-742.

Page, L. A., K. Rubia, Q. Deeley, E. Daly, F. Toal, D. Mataix-Cols, V. Giampietro, N. Schmitz and D. G. Murphy (2009). "A functional magnetic resonance imaging study of inhibitory control in obsessive-compulsive disorder." Psychiatry Research: Neuroimaging **174**(3): 202-209.

Peterson, B. S., M. J. Kane, G. M. Alexander, C. Lacadie, P. Skudlarski, H. C. Leung, J. May and J. C. Gore (2002). "An event-related functional MRI study comparing interference effects in the Simon and Stroop tasks." Brain Res Cogn Brain Res **13**(3): 427-440.

Pompei, F., J. Jogia, R. Tatarelli, P. Girardi, K. Rubia, V. Kumari and S. Frangou (2011). "Familial and disease specific abnormalities in the neural correlates of the Stroop Task in Bipolar Disorder." Neuroimage **56**(3): 1677-1684.

Purmann, S. and S. Pollmann (2015). "Adaptation to recent conflict in the classical color-word Stroop-task mainly involves facilitation of processing of task-relevant information." Frontiers in human neuroscience **9**.

Schmitz, N., K. Rubia, E. Daly, A. Smith, S. Williams and D. G. Murphy (2006). "Neural correlates of executive function in autistic spectrum disorders." Biological psychiatry **59**(1): 7-16.

Sebastian, A., C. Baldermann, B. Feige, M. Katzev, E. Scheller, B. Hellwig, K. Lieb, C. Weiller, O. Tüscher and S. Klöppel (2013). "Differential effects of age on subcomponents of response inhibition." Neurobiology of aging **34**(9): 2183-2193.

Song, Y. and Y. Hakoda (2015). "An fMRI study of the functional mechanisms of Stroop/reverse-Stroop effects." Behavioural brain research **290**: 187-196.

Spielberg, J. M., G. A. Miller, W. Heller and M. T. Banich (2015). "Flexible brain network reconfiguration supporting inhibitory control." Proceedings of the National Academy of Sciences **112**(32): 10020-10025.

vel Grajewska, B. Ż., E.-J. Sim, K. Hoenig, B. Herrnberger and M. Kiefer (2011). "Mechanisms underlying flexible adaptation of cognitive control: Behavioral and neuroimaging evidence in a flanker task." Brain research **1421**: 52-65.

Wittfoth, M., D. Buck, M. Fahle and M. Herrmann (2006). "Comparison of two Simon tasks: neuronal correlates of conflict resolution based on coherent motion perception." Neuroimage **32**(2): 921-929.

Zhu, D. C., R. T. Zacks and J. M. Slade (2010). "Brain activation during interference resolution in young and older adults: an fMRI study." Neuroimage **50**(2): 810-817.

***Action withholding***

Altshuler, L. L., et al. (2005). "Blunted activation in orbitofrontal cortex during mania: a functional magnetic resonance imaging study." Biological psychiatry **58**(10): 763-769.

Asahi, S., et al. (2004). "Negative correlation between right prefrontal activity during response inhibition and impulsiveness: a fMRI study." European Archives of Psychiatry and Clinical Neuroscience **254**(4): 245-251.

Baglio, F., et al. (2011). "Functional brain changes in early Parkinson's disease during motor response and motor inhibition." Neurobiology of Aging **32**(1): 115-124.

Baumeister, S., et al. (2014). "Sequential inhibitory control processes assessed through simultaneous EEG–fMRI." Neuroimage **94**: 349-359.

Behan, B., et al. (2015). "Right prefrontal and ventral striatum interactions underlying impulsive choice and impulsive responding." Human Brain Mapping **36**(1): 187-198.

Bellgrove, M. A., et al. (2004). "The functional neuroanatomical correlates of response variability: evidence from a response inhibition task." Neuropsychologia **42**(14): 1910-1916.

Berkman, E. T., et al. (2009). "Inhibitory spillover: Intentional motor inhibition produces incidental limbic inhibition via right inferior frontal cortex." Neuroimage **47**(2): 705-712.

Blasi, G., et al. (2006). "Brain regions underlying response inhibition and interference monitoring and suppression." European Journal of Neuroscience **23**(6): 1658-1664.

Borgwardt, S. J., et al. (2008). "Neural basis of Δ-9-tetrahydrocannabinol and cannabidiol: effects during response inhibition." Biological Psychiatry **64**(11): 966-973.

Braver, T. S., et al. (2001). "Anterior cingulate cortex and response conflict: effects of frequency, inhibition and errors." Cerebral Cortex **11**(9): 825-836.

Brosch, T., et al. (2011). "Generating value (s): Psychological value hierarchies reflect context-dependent sensitivity of the reward system." Social Neuroscience **6**(2): 198-208.

Brown, M. R., et al. (2006). "Inhibition and generation of saccades: rapid event-related fMRI of prosaccades, antisaccades, and nogo trials." Neuroimage **33**(2): 644-659.

Brown, M. R., et al. (2012). "Effects of emotional context on impulse control." Neuroimage **63**(1): 434-446.

Brown, M. R., et al. (2008). "Isolation of saccade inhibition processes: rapid event-related fMRI of saccades and nogo trials." Neuroimage **39**(2): 793-804.

Brown, S. M., et al. (2006). "Neural basis of individual differences in impulsivity: contributions of corticolimbic circuits for behavioral arousal and control." Emotion **6**(2): 239.

Bunge, S. A., et al. (2002). "Immature frontal lobe contributions to cognitive control in children: evidence from fMRI." Neuron **33**(2): 301-311.

Burke, M. R. and G. R. Barnes (2011). "The neural correlates of inhibiting pursuit to smoothly moving targets." Journal of Cognitive Neuroscience **23**(11): 3294-3303.

Chen, C. Y., et al. (2015). "Brain correlates of response inhibition in Internet gaming disorder." Psychiatry and Clinical Neurosciences **69**(4): 201-209.

Chiang, H.-S., et al. (2013). "Semantic processing and response inhibition." Neuroreport **24**(16): 889-893.

Chikazoe, J., et al. (2009). "Functional dissociation in right inferior frontal cortex during performance of go/no-go task." Cerebral Cortex **19**(1): 146-152.

Chuah, Y. L., et al. (2006). "The neural basis of interindividual variability in inhibitory efficiency after sleep deprivation." The Journal of Neuroscience **26**(27): 7156-7162.

De Zubicaray, G., et al. (2000). "Motor response suppression and the prepotent tendency to respond: a parametric fMRI study." Neuropsychologia **38**(9): 1280-1291.

Dillo, W., et al. (2010). "Neuronal correlates of ADHD in adults with evidence for compensation strategies–a functional MRI study with a Go/No-Go paradigm." GMS German Medical Science **8**.

Dodds, C. M., et al. (2010). "Dissociating inhibition, attention, and response control in the frontoparietal network using functional magnetic resonance imaging." Cerebral Cortex: bhq187.

Duerden, E. G., et al. (2013). "Neural correlates of inhibition of socially relevant stimuli in adults with autism spectrum disorder." Brain Research **1533**: 80-90.

Durston, S., et al. (2002). "The effect of preceding context on inhibition: an event-related fMRI study." Neuroimage **16**(2): 449-453.

Falconer, E., et al. (2008). "The neural networks of inhibitory control in posttraumatic stress disorder." Journal of Psychiatry & Neuroscience: JPN **33**(5): 413.

Fassbender, C., et al. (2009). "Prefrontal and midline interactions mediating behavioural control." European Journal of Neuroscience **29**(1): 181-187.

Fassbender, C., et al. (2004). "A topography of executive functions and their interactions revealed by functional magnetic resonance imaging." Cognitive Brain Research **20**(2): 132-143.

Fassbender, C., et al. (2006). "The role of a right fronto-parietal network in cognitive control." Journal of Psychophysiology **20**(4): 286-296.

Fedota, J. R., et al. (2014). "Representation of response alternatives in human presupplementary motor area: Multi-voxel pattern analysis in a go/no-go task." Neuropsychologia **56**: 110-118.

Garavan, H., et al. (2006). "Individual differences in the functional neuroanatomy of inhibitory control." Brain Research **1105**(1): 130-142.

Garavan, H., et al. (2003). "A midline dissociation between error-processing and response-conflict monitoring." Neuroimage **20**(2): 1132-1139.

Garavan, H., et al. (2002). "Dissociable executive functions in the dynamic control of behavior: inhibition, error detection, and correction." Neuroimage **17**(4): 1820-1829.

Goghari, V. M. and A. W. MacDonald (2009). "The neural basis of cognitive control: Response selection and inhibition." Brain and Cognition **71**(2): 72-83.

Goldstein, M., et al. (2007). "Neural substrates of the interaction of emotional stimulus processing and motor inhibitory control: an emotional linguistic go/no-go fMRI study." Neuroimage **36**(3): 1026-1040.

Goya-Maldonado, R., et al. (2010). "Motor impulsivity and the ventrolateral prefrontal cortex." Psychiatry Research: Neuroimaging **183**(1): 89-91.

Hare, T. A., et al. (2005). "Contributions of amygdala and striatal activity in emotion regulation." Biological Psychiatry **57**(6): 624-632.

Hester, R., et al. (2004). "Predicting success: patterns of cortical activation and deactivation prior to response inhibition." Journal of Cognitive Neuroscience **16**(5): 776-785.

Hester, R., et al. (2004). "Beyond common resources: the cortical basis for resolving task interference." Neuroimage **23**(1): 202-212.

Holz, N. E., et al. (2014). "Effect of prenatal exposure to tobacco smoke on inhibitory control: neuroimaging results from a 25-year prospective study." JAMA psychiatry **71**(7): 786-796.

Horn, N., et al. (2003). "Response inhibition and impulsivity: an fMRI study." Neuropsychologia **41**(14): 1959-1966.

Jacob, G. A., et al. (2013). "Emotional modulation of motor response inhibition in women with borderline personality disorder: an fMRI study." Journal of Psychiatry & Neuroscience: JPN **38**(3): 164.

Jamadar, S., et al. (2010). "The spatial and temporal dynamics of anticipatory preparation and response inhibition in task-switching." Neuroimage **51**(1): 432-449.

Kühn, S., et al. (2009). "Intentional inhibition: How the “veto‐area” exerts control." Human Brain Mapping **30**(9): 2834-2843.

Kaladjian, A., et al. (2007). "Blunted activation in right ventrolateral prefrontal cortex during motor response inhibition in schizophrenia." Schizophrenia Research **97**(1): 184-193.

Kaladjian, A., et al. (2009). "Reduced brain activation in euthymic bipolar patients during response inhibition: an event-related fMRI study." Psychiatry Research: Neuroimaging **173**(1): 45-51.

Kaladjian, A., et al. (2009). "Remission from mania is associated with a decrease in amygdala activation during motor response inhibition." Bipolar Disorders **11**(5): 530-538.

Karch, S., et al. (2008). "Influence of trait anxiety on inhibitory control in alcohol-dependent patients: simultaneous acquisition of ERPs and BOLD responses." Journal of Psychiatric Research **42**(9): 734-745.

Kelly, A., et al. (2004). "Prefrontal‐subcortical dissociations underlying inhibitory control revealed by event‐related fMRI." European Journal of Neuroscience **19**(11): 3105-3112.

Kiehl, K. A., et al. (2000). "Error processing and the rostral anterior cingulate: An event‐related fMRI study." Psychophysiology **37**(2): 216-223.

Ko, C.-H., et al. (2014). "Altered brain activation during response inhibition and error processing in subjects with Internet gaming disorder: a functional magnetic imaging study." European Archives of Psychiatry and Clinical Neuroscience **264**(8): 661-672.

Konishi, S., et al. (1999). "Common inhibitory mechanism in human inferior prefrontal cortex revealed by event-related functional MRI." Brain **122**(5): 981-991.

Konishi, S., et al. (1998). "No‐go dominant brain activity in human inferior prefrontal cortex revealed by functional magnetic resonance imaging." European Journal of Neuroscience **10**(3): 1209-1213.

Lütcke, H. and J. Frahm (2008). "Lateralized anterior cingulate function during error processing and conflict monitoring as revealed by high-resolution fMRI." Cerebral Cortex **18**(3): 508-515.

Langenecker, S. A., et al. (2007). "Frontal and limbic activation during inhibitory control predicts treatment response in major depressive disorder." Biological Psychiatry **62**(11): 1272-1280.

Laurens, K. R., et al. (2005). "A supramodal limbic‐paralimbic‐neocortical network supports goal‐directed stimulus processing." Human Brain Mapping **24**(1): 35-49.

Lawrence, E. J., et al. (2009). "The neural basis of response inhibition and attention allocation as mediated by gestational age." Human Brain Mapping **30**(3): 1038-1050.

Liddle, P. F., et al. (2001). "Event‐related fMRI study of response inhibition." Human Brain Mapping **12**(2): 100-109.

Maguire, R., et al. (2003). "Evidence of enhancement of spatial attention during inhibition of a visuo-motor response." Neuroimage **20**(2): 1339-1345.

Maltby, N., et al. (2005). "Dysfunctional action monitoring hyperactivates frontal–striatal circuits in obsessive–compulsive disorder: an event-related fMRI study." Neuroimage **24**(2): 495-503.

Mazzola-Pomietto, P., et al. (2009). "Bilateral decrease in ventrolateral prefrontal cortex activation during motor response inhibition in mania." Journal of Psychiatric Research **43**(4): 432-441.

McNab, F., et al. (2008). "Common and unique components of inhibition and working memory: an fMRI, within-subjects investigation." Neuropsychologia **46**(11): 2668-2682.

Menon, V., et al. (2001). "Error‐related brain activation during a Go/NoGo response inhibition task." Human Brain Mapping **12**(3): 131-143.

Mobbs, D., et al. (2007). "Frontostriatal dysfunction during response inhibition in Williams syndrome." Biological Psychiatry **62**(3): 256-261.

Mostofsky, S. H., et al. (2003). "fMRI evidence that the neural basis of response inhibition is task-dependent." Cognitive Brain Research **17**(2): 419-430.

Nakata, H., et al. (2008). "Executive functions with different motor outputs in somatosensory Go/Nogo tasks: an event-related functional MRI study." Brain Research Bulletin **77**(4): 197-205.

Nakata, H., et al. (2008). "Somato-motor inhibitory processing in humans: an event-related functional MRI study." Neuroimage **39**(4): 1858-1866.

Nakata, H., et al. (2009). "Negative BOLD effect on somato-motor inhibitory processing: an fMRI study." Neuroscience Letters **462**(2): 101-104.

O'Connor, D. A., et al. (2012). "Successful inhibitory control over an immediate reward is associated with attentional disengagement in visual processing areas." Neuroimage **62**(3): 1841-1847.

Roth, R. M., et al. (2007). "Event-related functional magnetic resonance imaging of response inhibition in obsessive-compulsive disorder." Biological Psychiatry **62**(8): 901-909.

Rubia, K., et al. (2001). "Mapping motor inhibition: conjunctive brain activations across different versions of go/no-go and stop tasks." Neuroimage **13**(2): 250-261.

Rubia, K., et al. (2007). "Linear age‐correlated functional development of right inferior fronto‐striato‐cerebellar networks during response inhibition and anterior cingulate during error‐related processes." Human Brain Mapping **28**(11): 1163-1177.

Rubia, K., et al. (2006). "Progressive increase of frontostriatal brain activation from childhood to adulthood during event‐related tasks of cognitive control." Human Brain Mapping **27**(12): 973-993.

Schulz, K. P., et al. (2009). "Dissociable neural effects of stimulus valence and preceding context during the inhibition of responses to emotional faces." Human Brain Mapping **30**(9): 2821-2833.

Sebastian, A., et al. (2012). "Neural correlates of interference inhibition, action withholding and action cancelation in adult ADHD." Psychiatry Research: Neuroimaging **202**(2): 132-141.

Sebastian, A., et al. (2013). "Disentangling common and specific neural subprocesses of response inhibition." Neuroimage **64**: 601-615.

Shafritz, K. M., et al. (2015). "Neural systems mediating decision-making and response inhibition for social and nonsocial stimuli in autism." Progress in Neuro-Psychopharmacology and Biological Psychiatry **60**: 112-120.

Steele, V. R., et al. (2013). "A large scale (N= 102) functional neuroimaging study of response inhibition in a Go/NoGo task." Behavioural Brain Research **256**: 529-536.

Tamm, L., et al. (2004). "Event-related FMRI evidence of frontotemporal involvement in aberrant response inhibition and task switching in attention-deficit/hyperactivity disorder." Journal of the American Academy of Child & Adolescent Psychiatry **43**(11): 1430-1440.

Todd, R. M., et al. (2012). "Withholding response in the face of a smile: age-related differences in prefrontal sensitivity to Nogo cues following happy and angry faces." Developmental Cognitive Neuroscience **2**(3): 340-350.

Townsend, J. D., et al. (2012). "Deficits in inferior frontal cortex activation in euthymic bipolar disorder patients during a response inhibition task." Bipolar Bisorders **14**(4): 442-450.

Völlm, B., et al. (2004). "Neurobiological substrates of antisocial and borderline personality disorder: preliminary results of a functional fMRI study." Criminal Behaviour and Mental Health **14**(1): 39-54.

van Gaal, S., et al. (2010). "Unconscious activation of the prefrontal no-go network." The Journal of Neuroscience **30**(11): 4143-4150.

Vanderhasselt, M.-A., et al. (2011). "Healthy brooders employ more attentional resources when disengaging from the negative: an event-related fMRI study." Cognitive, Affective, & Behavioral Neuroscience **11**(2): 207-216.

Vercammen, A., et al. (2012). "Reduced neural activity of the prefrontal cognitive control circuitry during response inhibition to negative words in people with schizophrenia." Journal of Psychiatry & Neuroscience: JPN **37**(6): 379.

Wager, T. D., et al. (2005). "Common and unique components of response inhibition revealed by fMRI." Neuroimage **27**(2): 323-340.

Walther, S., et al. (2010). "A supramodal network for response inhibition." Neuroreport **21**(3): 191-195.

Watanabe, J., et al. (2002). "The human prefrontal and parietal association cortices are involved in NO-GO performances: an event-related fMRI study." Neuroimage **17**(3): 1207-1216.

Welander‐Vatn, A. S., et al. (2009). "No altered dorsal anterior cingulate activation in bipolar II disorder patients during a Go/No‐go task: an fMRI study." Bipolar Disorders **11**(3): 270-279.

Zheng, D., et al. (2008). "The key locus of common response inhibition network for no-go and stop signals." Journal of Cognitive Neuroscience **20**(8): 1434-1442.

Bernal, B. and N. Altman (2009). "Neural networks of motor and cognitive inhibition are dissociated between brain hemispheres: an fMRI study." International Journal of Neuroscience **119**(10): 1848-1880.

Booth, J. R., D. D. Burman, J. R. Meyer, Z. Lei, B. L. Trommer, N. D. Davenport, W. Li, T. B. Parrish, D. R. Gitelman and M. M. Mesulam (2003). "Neural development of selective attention and response inhibition." Neuroimage **20**(2): 737-751.

Chen, C.-Y., J.-Y. Yen, C.-F. Yen, C.-S. Chen, G.-C. Liu, C.-Y. Liang and C.-H. Ko (2015). "Aberrant brain activation of error processing among adults with attention deficit and hyperactivity disorder." The Kaohsiung journal of medical sciences **31**(4): 179-187.

Cojan, Y., L. Waber, A. Carruzzo and P. Vuilleumier (2009). "Motor inhibition in hysterical conversion paralysis." Neuroimage **47**(3): 1026-1037.

Del-Ben, C. M., J. W. Deakin, S. Mckie, N. A. Delvai, S. R. Williams, R. Elliott, M. Dolan and I. M. Anderson (2005). "The effect of citalopram pretreatment on neuronal responses to neuropsychological tasks in normal volunteers: an FMRI study." Neuropsychopharmacology **30**(9): 1724-1734.

Hester, R. and H. Garavan (2004). "Executive dysfunction in cocaine addiction: evidence for discordant frontal, cingulate, and cerebellar activity." The Journal of Neuroscience **24**(49): 11017-11022.

Jamadar, S., M. Hughes, W. Fulham, P. Michie and F. Karayanidis (2010). "The spatial and temporal dynamics of anticipatory preparation and response inhibition in task-switching." Neuroimage **51**(1): 432-449.

Kaufman, J. N., T. J. Ross, E. A. Stein and H. Garavan (2003). "Cingulate hypoactivity in cocaine users during a GO-NOGO task as revealed by event-related functional magnetic resonance imaging." J Neurosci **23**(21): 7839-7843.

Langenecker, S. A. and K. A. Nielson (2003). "Frontal recruitment during response inhibition in older adults replicated with fMRI." Neuroimage **20**(2): 1384-1392.

Mobbs, D., M. A. Eckert, D. Mills, J. Korenberg, U. Bellugi, A. M. Galaburda and A. L. Reiss (2007). "Frontostriatal dysfunction during response inhibition in Williams syndrome." Biological Psychiatry **62**(3): 256-261.

Nielson, K. A., S. A. Langenecker and H. Garavan (2002). "Differences in the functional neuroanatomy of inhibitory control across the adult life span." Psychology and Aging **17**(1): 56-71.

O'Connor, D. A., D. J. Upton, J. Moore and R. Hester (2014). "Motivationally significant self-control: Enhanced action withholding involves the right inferior frontal junction." Journal of cognitive neuroscience.

Page, L. A., K. Rubia, Q. Deeley, E. Daly, F. Toal, D. Mataix-Cols, V. Giampietro, N. Schmitz and D. G. Murphy (2009). "A functional magnetic resonance imaging study of inhibitory control in obsessive-compulsive disorder." Psychiatry Research: Neuroimaging **174**(3): 202-209.

Peduto, A. and G. O. AssDip (2008). "The neural networks of inhibitory control in posttraumatic stress disorder." Journal of psychiatry & neuroscience: JPN **33**(5): 413.

Penfold, C., N. Vizueta, J. D. Townsend, S. Y. Bookheimer and L. L. Altshuler (2015). "Frontal lobe hypoactivation in medication-free adults with bipolar II depression during response inhibition." Psychiatry Research: Neuroimaging **231**(3): 202-209.

Rothmayr, C., B. Sodian, G. Hajak, K. Döhnel, J. Meinhardt and M. Sommer (2011). "Common and distinct neural networks for false-belief reasoning and inhibitory control." Neuroimage **56**(3): 1705-1713.

Schmitz, N., K. Rubia, E. Daly, A. Smith, S. Williams and D. G. Murphy (2006). "Neural correlates of executive function in autistic spectrum disorders." Biological psychiatry **59**(1): 7-16.

Schulz, K. P., A.-C. V. Bédard, R. Czarnecki and J. Fan (2011). "Preparatory activity and connectivity in dorsal anterior cingulate cortex for cognitive control." Neuroimage **57**(1): 242-250.

Sebastian, A., C. Baldermann, B. Feige, M. Katzev, E. Scheller, B. Hellwig, K. Lieb, C. Weiller, O. Tüscher and S. Klöppel (2013). "Differential effects of age on subcomponents of response inhibition." Neurobiology of aging **34**(9): 2183-2193.

Shane, M. S., M. Stevens, C. L. Harenski and K. A. Kiehl (2008). "Neural correlates of the processing of another’s mistakes: a possible underpinning for social and observational learning." Neuroimage **42**(1): 450-459.

Singh, M. K., K. D. Chang, P. Mazaika, A. Garrett, N. Adleman, R. Kelley, M. Howe and A. Reiss (2010). "Neural correlates of response inhibition in pediatric bipolar disorder." Journal of Child and Adolescent Psychopharmacology **20**(1): 15-24.

Talati, A. and J. Hirsch (2005). "Functional specialization within the medial frontal gyrus for perceptual go/no-go decisions based on “what,”“when,” and “where” related information: an fMRI study." Journal of cognitive neuroscience **17**(7): 981-993.

Vidal, J., T. Mills, E. W. Pang and M. J. Taylor (2012). "Response inhibition in adults and teenagers: spatiotemporal differences in the prefrontal cortex." Brain and cognition **79**(1): 49-59.

Welander-Vatn, A., J. Jensen, M. K. Otnaess, I. Agartz, A. Server, I. Melle and O. A. Andreassen (2013). "The neural correlates of cognitive control in bipolar I disorder: an fMRI study of medial frontal cortex activation during a Go/No-go task." Neuroscience letters **549**: 51-56.

Wen, T. and S. Hsieh (2015). "Neuroimaging of the joint Simon effect with believed biological and non-biological co-actors." Frontiers in human neuroscience **9**.

Ye, Z., E. Altena, C. Nombela, C. R. Housden, H. Maxwell, T. Rittman, C. Huddleston, C. L. Rae, R. Regenthal and B. J. Sahakian (2015). "Improving response inhibition in Parkinson’s disease with atomoxetine." Biological psychiatry **77**(8): 740-748.

***Action cancellation***
